# Widespread Phenological Shifts With Temperature in Alaska's Marine Fishes

**DOI:** 10.1111/gcb.70708

**Published:** 2026-01-16

**Authors:** Lauren A. Rogers, Kelia E. Axler, Jennifer S. Bigman

**Affiliations:** ^1^ Alaska Fisheries Science Center, National Marine Fisheries Service, National Oceanic and Atmospheric Administration Seattle Washington USA; ^2^ Office of Science and Technology, National Marine Fisheries Service, National Oceanic and Atmospheric Administration Seattle Washington USA; ^3^ School of Marine Science and Policy University of Delaware Lewes Delaware USA

**Keywords:** Alaska, ichthyoplankton, larval fish, match‐mismatch, phenology, size‐at‐date, spatiotemporal models, spawning

## Abstract

Changes in the timing of fish spawning and early life stage development can affect the temporal match or mismatch of larvae with production of preferred prey as well as their availability to predators, with potential consequences for recruitment success, food‐web dynamics, and fisheries. Using > 370,000 observations from over four decades of spring ichthyoplankton surveys in the Gulf of Alaska and Bering Sea, we investigated long‐term changes in the phenology of 29 fish species, including commercially important taxa such as Pacific cod, walleye pollock, and Pacific halibut. Larval size on a standardized date (size‐at‐date) was used as a proxy for larval developmental timing in spring, and reflects a combination of hatch timing (larval age), growth, and mortality. Spatiotemporal generalized linear mixed models were used to account for variable sampling effort in space and time in order to isolate long‐term trends and thermal effects on larval size. For a majority of species, interannual variation in mean size‐at‐date was significantly and positively related to temperature, demonstrating widespread thermal effects on the phenology of fish early life stages. Despite the wide diversity of life history traits exhibited by the 29 species examined, patterns in size‐at‐date over time were similar across most species within each ecosystem, reflecting the common effect of temperature on phenology. While temperature affected size‐at‐date, there was little evidence of long‐term trends, likely due to the lack of a linear trend in winter–spring temperatures observed in recent decades. We demonstrate a novel analytical method to assess changes in phenology from larval size observations sampled at variable locations and times, and detect phenological shifts that were not necessarily identifiable from larval abundance data alone. Our results suggest that earlier spring phenology due to warming will be a common response among fishes to projected future climate change in high‐latitude ecosystems.

## Graphical abstract

We investigated changes in the phenology of early life stages of fishes in the Gulf of Alaska and Bering Sea. Larval size on a standardized date was used as a proxy for larval developmental timing in spring. For most species, interannual variation in mean size‐at‐date was significantly and positively related to temperature, demonstrating widespread thermal effects on the phenology of fish early life stages. Despite variable survey coverage and timing, the use of spatiotemporal models allowed us to draw inferences across 29 fish species, resulting in the most comprehensive analysis of changing fish phenology in these systems to date.

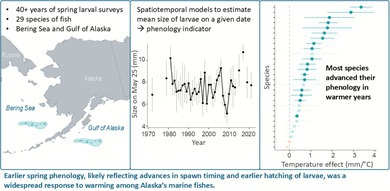

## 1 Introduction

Changes in phenology, or the seasonal timing of life‐history events, are a commonly observed response to changes in climate (Poloczanska et al. 2013), and may alter predator–prey interactions, community dynamics, and population productivity. Warming is generally expected to advance springtime phenology, but this does not appear to be universal across species and systems. Some species have a more fixed timing of life history events while others delay their timing under warming (Thackeray et al. 2010). These differences in response to warming reflect the diversity of physiological processes and behavioral mechanisms underlying changes in phenology within and across species (Chmura et al. 2019). Understanding the sensitivity of phenology to warming is an important, yet complex aspect of studying adaptation to climate change. Marine systems in particular remain understudied with respect to phenology, due in part to challenges associated with observing seasonal events with ship‐based sampling. This has limited our understanding of phenological dynamics in the marine realm, particularly for fishes (Rademaker et al. 2024; Dugan et al. 2024). Given historical and future projected warming in marine ecosystems (Venegas et al. 2023), detecting and understanding patterns of phenological responses will be important for anticipating future ecosystem dynamics and changes in ecosystem services, such as the provisioning of wild fish.

In temperate to high‐latitude systems, the spawning of marine fishes is often highly seasonal, timed such that newly‐hatched offspring coincide with the increase in prey production during spring. After hatching from eggs, survival depends on first‐feeding larvae finding sufficient prey (often microzooplankton) once their intrinsic yolk‐sac reserves are exhausted. A mismatch in the timing of these processes has been invoked to explain recruitment patterns per the match‐mismatch hypothesis of Cushing (1990). Even beyond the first‐feeding period, the first few weeks of life encompass a critical period for fishes (Hjort 1914) when mortality rates are high. Small changes in the timing of spawning and hatching, as well as growth rates, can affect the size of larvae obtained by a given date, impacting their ability to capture and ingest larger prey, evade predators, outcompete competitors, and recruit to the adult population (Houde 1987; Anderson 1988; Miller et al. 1988; Fennie et al. 2024). Life history transitions, such as the transformation from pre‐ to post‐flexion developmental stages, or settlement from pelagic to demersal habitat, are often size‐dependent (Matarese 1989; Fedewa et al. 2016). Therefore, timing and size are linked, particularly for fish early life stages (Miller et al. 2024), such that changes in larval size obtained by a given date are indicative of species phenology.

Studying changes in fish spawn timing and early life stage development typically requires detailed laboratory work and/or extensive sampling in space and time. For instance, daily increments in otoliths from larval and juvenile fish have been used to assess changes in the timing of spawning and hatching, but only for a limited number of species (e.g., Woodbury and Ralston 1991; Fox et al. 2007; Rogers and Dougherty 2019; Almeida et al. 2024). Such studies have documented the effects of environmental conditions on fish spawn timing, as well as interannual differences in the timing of larval first‐feeding, juvenile metamorphosis, or settlement to benthic habitats (e.g., Fedewa et al. 2016; Raventos et al. 2021; Vaz et al. 2023). However, reading larval and juvenile otoliths is highly labor‐intensive, and as a result, long time series of otolith‐derived hatch dates are rare in the literature. In addition, not all species deposit daily growth increments in their otoliths, which further limits the use of this approach for broader multispecies studies. Alternatively, changes in the timing of seasonal peaks in egg or larval abundance observed in the field have been used to infer changes in spawn timing and early life stage phenology (e.g., Greve et al. 2005; Genner et al. 2010; Asch 2015; Auth et al. 2018; Weisberg et al. 2024; Chen et al. 2025). For instance, Asch (2015) found that the central tendency (median month of occurrence) of fish larvae shifted ~1 week earlier in recent decades in the southern California Current. This “central tendency” approach depends on repeated surveys in the same geographic area throughout a season or year, and is analogous to approaches in terrestrial systems where repeated seasonal observations are more common, for example, when estimating timing of peak flowering. However, such sampling is costly, particularly in offshore environments, and temporal coverage is often limited. This is particularly an issue for species that are present in the plankton for a relatively contracted period, requiring monthly or even weekly resampling to capture changes in timing.

In this study, we use measurements of larval size to gain insight into changes in the phenology of development in marine fishes. Particularly for species with seasonal spawning windows, larval size distributions collected on a single survey in a year can provide a snapshot of the relative stage of larval development obtained by the survey date. Individual larval size is a function of age (the difference between hatch date and sampling date) and growth rate. At a population level, larval size distributions represent the range of hatch dates and growth rates in the population, as well as accumulated mortality, which may be size‐selective. As such, larval size contains information about the current developmental stage of larvae, as well as a window into their environmental experience during the first days and weeks of life. Larval size can also contain information about the environmental experience of parents (e.g., Rogers and Dougherty 2019; Fennie et al. 2024), as parental spawn timing may depend on thermal or other conditions. Because larval size is highly dependent on age and thus the timing (day) of sampling, any attempt to estimate phenological responses from larval size observations must account for sampling day, which may vary both within a survey and among years. We present a method using spatiotemporal models that allows us to account for differences in sampling time and location to draw inferences about larval size obtained on a given date each year.

We analyzed four decades of larval size observations from 29 marine fish species in the Gulf of Alaska and Bering Sea to investigate species‐specific and community‐level patterns of phenology. Specifically, we asked: (1) How variable is larval size‐at‐date from year to year (i.e., is there interannual variation in phenology)?; (2) Do species exhibit similar temporal patterns in size‐at‐date? In other words, have species shifted their phenology synchronously?; (3) Is larval size‐at‐date related to thermal conditions, with the expectation that warmer conditions will lead to earlier spring phenology and therefore larger size on a given date?; and (4) Have there been long‐term trends in size‐at‐date indicative of a long‐term phenological shift?

## 2 Methods

### 2.1 Study Region

The Eastern Bering Sea (EBS) and Gulf of Alaska (GOA) large marine ecosystems (LMEs) are high‐latitude marine ecosystems which support productive fisheries and have experienced large climate fluctuations in recent years. The EBS is a broad, shallow shelf ecosystem, and dynamics are largely regulated by the seasonal extent of sea ice (Stabeno et al. 2012), which has been decreasing over the long term and reached a record low in 2018 (Stabeno and Bell 2019). The GOA is a narrower shelf ecosystem, intersected by sea valleys and islands, and remains ice‐free year round. From 2014 to 2016, and again in 2019, marine heatwaves in the GOA resulted in fish stock declines and persistent shifts in lower to upper trophic level organisms (Barbeaux et al. 2020; Suryan et al. 2021). Such fluctuations make these ideal study systems for investigating changes in phenology and their link to environmental conditions.

### 2.2 Survey Data

**FIGURE 1:**
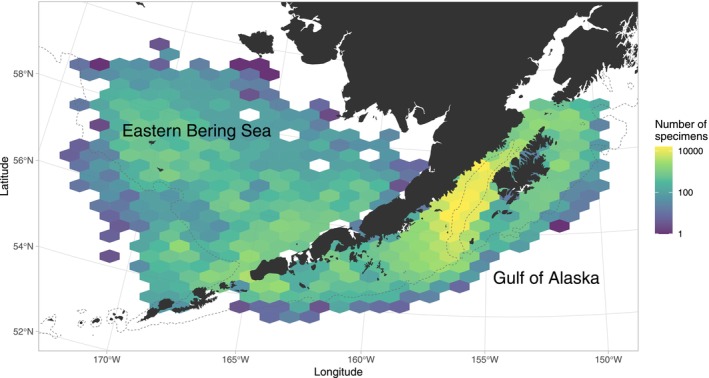
Map of study area in the western Gulf of Alaska and Eastern Bering Sea. Sampling intensity is indicated by the total number of specimens collected in each hexagon from 1972 to 2022. Dotted lines show the 200 m isobath.

Ichthyoplankton surveys in Alaska's waters have been conducted by the National Oceanic and Atmospheric Administration's Alaska Fisheries Science Center (NOAA AFSC) and partners since the 1970s, becoming more frequent in the 1980s (GOA) and 1990s (EBS). At each survey station, paired 60‐cm bongo nets were towed obliquely from a depth of at least 100 m, or 10 m off the bottom in shallower waters. Net mesh sizes were either 333‐ or 505‐μm. For each tow, the contents of one net were preserved in 5% formalin and later sorted and identified to the lowest taxonomic level possible at the Plankton Sorting and Identification Center in Szczecin, Poland. Up to 50 larvae of each taxa from each tow were measured for standard length after preservation. Species identifications were verified by expert taxonomists at NOAA AFSC. Survey coverage and timing have varied over the years due to differing survey objectives and ship time allocations (Figure S1), although many earlier surveys were specifically targeted for studying pollock eggs and larvae. For this analysis, we selected study areas that represented the most consistently sampled areas within each region (Figure 1) and used only samples collected within the most consistently sampled temporal window (Days 98–162 in the EBS and 117–160 in the GOA). This spring (April–June) sampling window captures a high diversity and abundance of larval fishes, reflecting common patterns of winter–spring spawning in these systems (Matarese et al. 2003; Doyle et al. 2019). Larval size data are available on Dryad at https://doi.org/10.5061/dryad.2280gb66b (Rogers et al. 2026).

### 2.3 Species Selection

Only taxa identified to the species level were included for analysis. From those, we chose species that were present in at least 10 different years, with at least 10 observations in each of those years. Observations from the two LMEs were considered separately for species selection. Four species in the GOA and five species in the EBS were further excluded because models consistently failed to converge, likely due to sparse sample coverage. This resulted in the inclusion of 29 species overall, with 28 species from the Gulf of Alaska and six species from the Bering Sea; five species were included from both LMEs (Table 1). Observations for a species in years when fewer than 10 specimens of that species were measured were excluded. Available life history traits and ecological information (e.g., longevity, fecundity, spawning habitat and timing, egg type, pelagic larval duration) for these species were compiled from a variety of sources including Doyle et al. (2019), Marshall et al. (2019), Matarese (1989), and Matarese et al. (2003), summarized in Table S1.

### 2.4 Environmental Data

For the Bering Sea, the thermal environment was characterized using depth‐averaged water column temperatures from the Bering10K ROMS hindcast for the Bering Sea (Hermann et al. 2021). Depth‐averaged temperatures were used rather than surface or bottom temperatures to capture the variable habitat used by species for spawning and egg and larval development. Depth‐averaged temperatures were spatially averaged over the SE Bering Sea shelf as in Kearney (2021), and then averaged by month. In the Gulf of Alaska, historical water column temperatures were not available over the time‐scale of this study; thus, we used monthly sea surface temperatures (SST) from the NCEP reanalysis (Kalnay et al. 1996) for a region in the western Gulf of Alaska defined by 54.3° N to 56.2° N and 151.9° W to 157.5° W. Particularly in winter months when the water column over the shelf is well‐mixed, variation in SST is highly correlated with temperatures at depth (Danielson et al. 2022).

### 2.5 Statistical Modeling Framework

We used spatiotemporal generalized linear mixed models (GLMMs) to investigate changes in larval size through time and as a function of temperature, addressing each of our four study questions introduced above. This approach allowed us to account, as best as possible, for differences in sampling gear (i.e., mesh size), day of year, and location. All models included a fixed effect of mesh size as a factor to account for potential differences in size of larvae captured by the 333‐ and 505‐μm nets. Day of year of sampling was included as a smooth term (penalized spline) to account for changes in mean size through the sampling season. Latent spatiotemporal variation was modeled with independent and identically distributed spatiotemporal Gaussian random fields. These random fields (one for each year) capture variation in size that is spatially correlated but not captured by other model covariates. Separate models for each species and region were implemented using the R package *sdmTMB* (Anderson et al. 2022).

**TABLE 1:** Gulf of Alaska (*n* = 28) and Bering Sea (*n* = 6) fish species included in the study.

Species	Common name	First year	Last year	*n* years	*n* specimens	Mean length (mm)	Length range (5–95 pctl, mm)
Gulf of Alaska							
*Ammodytes personatus*	Pacific sand lance	1972	2022	40	44,022	14.6	(7.9–24.5)
*Anoplarchus insignis*	Slender cockscomb	1985	2021	22	1339	9.7	(6.9–14.2)
*Anoplarchus purpurescens*	High cockscomb	1993	2019	12	308	8.4	(6.4–12.7)
*Artedius harringtoni*	Scalyhead sculpin	1981	2021	22	672	4.8	(3–7.8)
***Atheresthes stomias***	**Arrowtooth flounder**	1984	2022	30	6340	12.8	(7.8–19)
*Bathyagonus alascanus*	Gray starsnout	1981	2021	33	1745	8.3	(5.8–11.9)
***Clupea pallasii***	**Pacific herring**	1981	2021	16	877	11.1	(8–15.6)
*Cryptacanthodes aleutensis*	Dwarf Wrymouth	1981	2021	32	1411	15.8	(12.5–22)
***Gadus chalcogrammus***	**Walleye pollock**	1972	2021	39	147,303	7.3	(4.2–12.1)
***Gadus macrocephalus***	**Pacific cod**	1972	2021	39	18,021	6.5	(4–11)
***Glyptocephalus zachirus***	**Rex sole**	1981	2017	18	737	9.0	(7.1–11)
*Hexagrammos decagrammus*	Kelp greenling	1981	2021	28	1460	12.7	(9.9–16.6)
***Hippoglossoides elassodon***	**Flathead sole**	1979	2021	36	32,260	7.0	(4.9–10.4)
***Hippoglossus stenolepis***	**Pacific halibut**	1985	2021	22	1024	18.1	(14.5–23)
***Isopsetta isolepis***	**Butter sole**	1981	2019	14	1763	4.6	(3–7)
***Lepidopsetta bilineata***	**Southern rock sole**	1981	2021	30	4061	5.4	(3.2–9.8)
***Lepidopsetta polyxystra***	**Northern rock sole**	1972	2022	39	9122	7.2	(4–12)
*Leptoclinus maculatus*	Daubed shanny	1972	2019	24	1034	16.6	(11–24.8)
*Leuroglossus schmidti*	Northern smoothtongue	1981	2017	17	492	14.7	(9–22.3)
*Liparis fucensis*	Slipskin snailfish	1985	2021	17	898	4.9	(3.1–7.5)
***Microstomus pacificus***	**Dover sole**	1990	2017	10	514	7.4	(6.1–9)
***Platichthys stellatus***	**Starry flounder**	1981	2021	29	4077	4.1	(2.7–6.2)
***Pleuronectes quadrituberculatus***	**Alaska plaice**	1981	2013	16	381	6.2	(4.5–8.7)
*Poroclinus rothrocki*	Whitebarred prickleback	1981	2021	28	1177	14.3	(11.1–20)
*Radulinus asprellus*	Slim sculpin	1981	2019	22	600	6.8	(4.1–11)
*Ruscarius meanyi*	Puget sound sculpin	1992	2017	14	307	5.3	(3.3–8.4)
*Stenobrachius leucopsarus*	Northern lampfish	1981	2022	36	6494	5.6	(4–7.2)
*Zaprora silenus*	Prowfish	1991	2021	15	291	13.6	(10.6–18.4)
Bering Sea							
***Gadus chalcogrammus***	**Walleye pollock**	1979	2021	29	58,780	6.6	(4–11.5)
***Gadus macrocephalus***	**Pacific cod**	1991	2021	23	3310	6.4	(3.8–10)
***Hippoglossoides elassodon***	**Flathead sole**	1979	2018	11	6997	6.5	(4.5–9.1)
***Hippoglossus stenolepis***	**Pacific halibut**	1992	2017	13	390	16	(13.1–19)
***Lepidopsetta polyxystra***	**Northern rock sole**	1979	2021	29	12,477	5.7	(3.5–9.8)
***Reinhardtius hippoglossoides***	**Greenland halibut**	1991	2012	11	540	18.6	(15–22)

*Note:* Species in bold are commercially harvested. Species‐specific early life history traits are given in Table S1.

Starting with the model structure described above, three separate model formulations were fitted to address our research objectives. First, we investigated interannual changes in mean size (Question 1) by fitting models with year as a factor and extracting conditional year effects for a 505‐μm sampling mesh and Day 145 (May 25), which was the median sampling date for the GOA and within the larval phase for all species examined in this study (Table S1, Figure S2). These estimates of conditional mean size by species and year (larval size‐at‐date) were subsequently used to address Question 2 by conducting a multispecies analysis to compare length dynamics through time (see *Multispecies Comparison* below). Next, for each species, we tested whether there was an effect of temperature on larval size (Question 3) by fitting models with a fixed effect of mean January–May (JFMAM) temperature. This period represents the thermal environment in the 5 months leading up to sampling, which, for most species, covered periods associated with pre‐spawning, spawning, egg incubation, yolk‐sac larval development, and larval first feeding (Table S1). Finally, we tested whether there were long‐term linear trends in size‐at‐date (Question 4) by fitting models with a fixed effect of year as a continuous variable, excluding any temperature covariates.

We investigated several families of distributions for the larval size models, including gaussian, lognormal, and generalized gamma distributions. Parameter estimates and significance levels were similar across distributions; however, model convergence was most successful for the gaussian models, so we present those here. Models with a positive‐definite Hessian matrix and a maximum absolute log likelihood gradient < 0.001 were considered to have converged. Residuals were evaluated using the *DHARMa* package in R (Hartig 2022).

### 2.6 Multispecies Comparison

To investigate similarities in larval size‐at‐date across species through time (Question 2), we extracted the conditional year effects from the models with year as a factor, as described above. We then used Bayesian Dynamic Factor Analysis (DFA) to estimate shared trends, or interannual patterns, in larval size‐at‐date among species, and species‐specific loadings on those trends. Time‐series of larval size‐at‐date were standardized (Z‐scored) prior to analysis. Models with a single common trend, modeled as a random walk, were fitted to data from each region separately using the R package *bayesdfa* (Ward et al. 2024). For each model, we ran four MCMC chains for 4000 iterations each and discarded the first 2000 iterations as a warm‐up period, for a total of 8000 posterior samples. Shared trends for each region were then regressed against temperature covariates to investigate whether temperature was a common driver of larval size‐at‐date across species. Finally, to test whether estimated shared trends were sensitive to the inclusion of shorter time‐series, we refitted the DFA for the GOA to different subsets of species, with minimum time‐series cutoffs of 10 years (current threshold), 15, 20, and 25 years of observations, and compared the estimated trends.

### 2.7 Long‐Term Temperature Trends

Linear trends in temperature were investigated by regressing JFMAM temperature on year in each region. Analyses were done for all years (1972–2022), as well as for only the subset of years with data included in this study.

### 2.8 Testing Sensitivity to Small Sample Sizes

A sensitivity test was used to determine the ability of our models to estimate effects of temperature on larval size when limited to only 10 observations per year (our selected cutoff). Specifically, we used observations from the most data‐rich species, GOA walleye pollock, and randomly sampled 10 observations per year to create a minimal dataset. We then fit the spatiotemporal model with JFMAM temperature as a covariate and extracted the estimate of the temperature effect. We repeated this for 200 random subsets of data and compared the distribution of estimates from the minimal datasets to the estimate from the full dataset.

## 3 Results

We analyzed a total of 288,730 larval lengths from 6571 hauls in the Gulf of Alaska and 82,495 larval lengths from 2438 hauls in the Bering Sea, spanning the period 1972 to 2022 (Figure S1). Taxa included commercially important species, such as walleye pollock, Pacific cod, and Pacific halibut, as well as lesser‐studied species such as the Pacific blacksmelt and kelp greenling. Forage fish species such as Pacific sand lance and daubed shanny, as well as flatfishes such as Alaska plaice, flathead sole, rex sole, and northern and southern rock sole, were also represented in the larval data set. The most frequently occurring species was walleye pollock for both regions. While all species necessarily had larvae available to the sampling gear in spring, they varied in their spawning location, timing and duration, the spawning habitat or egg type (e.g., demersal, pelagic), and pelagic larval duration (Table S1).

**FIGURE 2:**
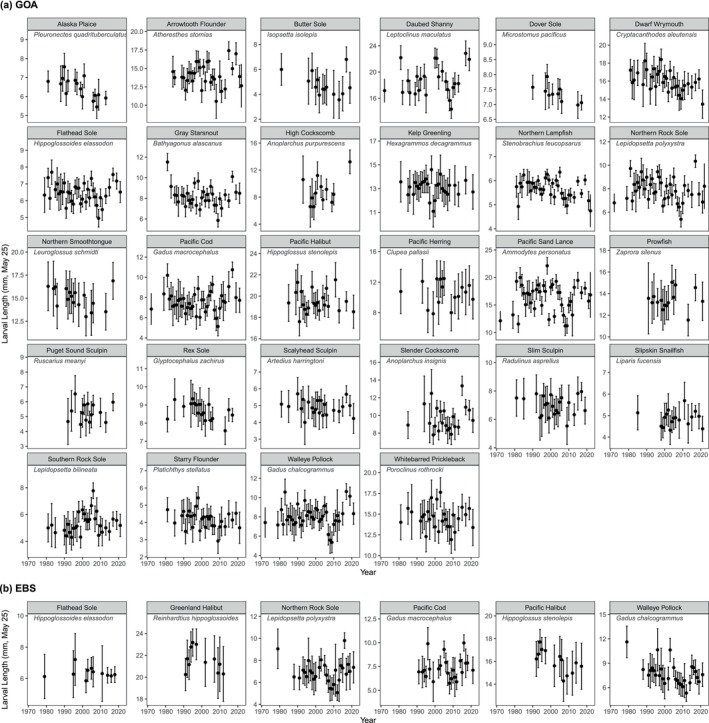
Estimated mean larval lengths by year with 95% CIs. Estimates are derived from models with year as a factor, conditioned on day of year 145 and mesh size 505‐μm for the (a) Gulf of Alaska and (b) Eastern Bering Sea.

**FIGURE 3:**
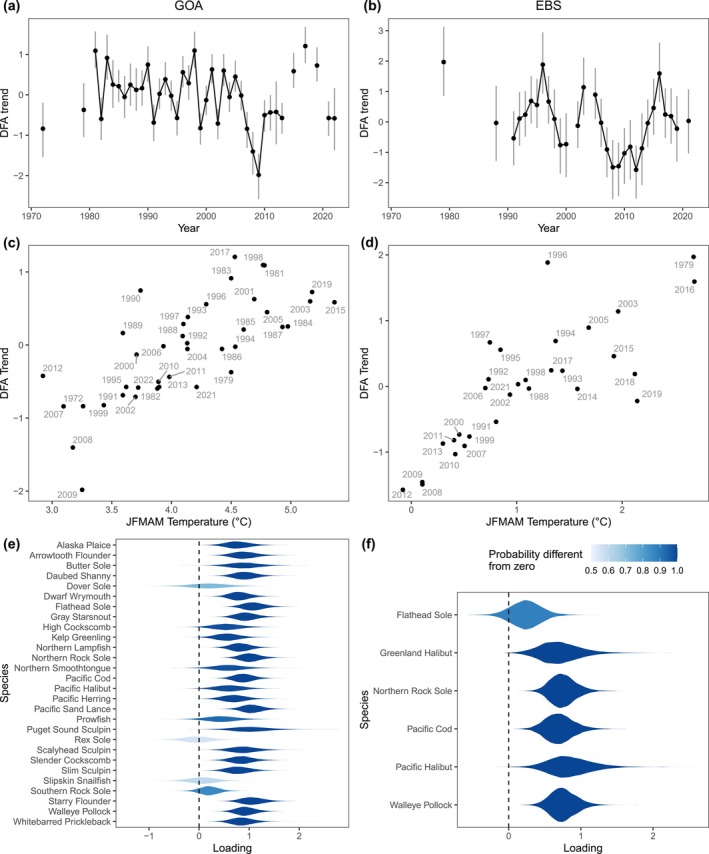
Results from Dynamic Factor Analysis (DFA) for the GOA (left) and EBS (right) showing (a, b) common trends in larval size‐at‐date (with 95% CIs), (c, d) their relationship with mean Jan‐May (JFMAM) temperatures, and (e, f) species‐specific loadings on those trends (violin plots show posterior densities).

Larval size‐at‐date varied from year to year for all 29 species examined and in both ecosystems (Figure 2). For instance, for Pacific sand lance in the GOA, mean length on May 25 was estimated to vary from 11 mm to 22 mm depending on the year, representing a twofold difference (Figure 2). Other species varied less in mean length, such as the 11–14 mm range in kelp greenling, or 6–8 mm range in Alaska plaice. Within ecosystems, common patterns in larval size‐at‐date were evident. On visual inspection, many GOA species experienced a notable decline in size‐at‐date during the 2000s with a minimum around 2009, and a rapid increase through the 2010s (Figure 2a). Shared patterns in the EBS were less visually apparent due to fewer years of data, although similarities can be noted among walleye pollock, Pacific cod, and northern rock sole (Figure 2b). These common trends were reflected in the DFA for each region (Figure 3). In the GOA, the shared trend estimated from DFA was highly variable through 2006, then indicated relatively small size‐at‐date for 2007–2013, followed by a period of larger size‐at‐date during 2015–2019. Most species loaded significantly positively on this trend, while only three (rex sole, Dover sole, and slipskin snailfish) had no or only a weak loading on this trend. The estimated trend was relatively insensitive to the inclusion criterion used to select species, with similar trends estimated for subsets of species sampled in only 10 years versus 15, 20, or 25 years (Figure S3). In the EBS, the common trend estimated from the DFA indicated a general pattern of larger size‐at‐date in 1994–1997, 2003–2005, and 2016, and an extended period of smaller size‐at‐date during 2007–2013. All six species loaded positively on the common trend, and all but flathead sole had significant loadings.

**FIGURE 4:**
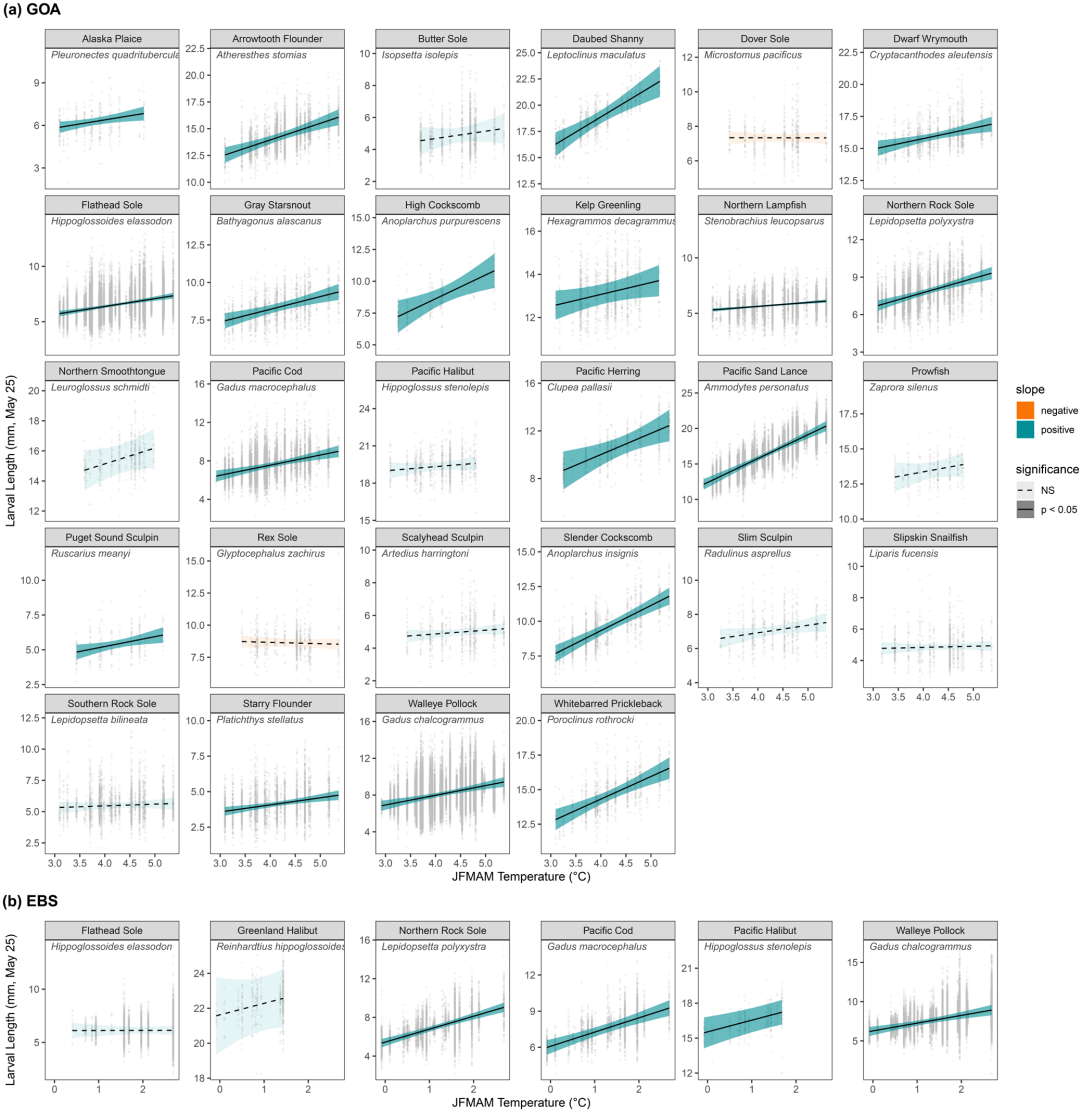
Conditional effect of JFMAM temperature on larval lengths, conditioned on day of year 145 and mesh size 505‐μm, for the (a) Gulf of Alaska and (b) Eastern Bering Sea. Significance (shading) is based on a 95% CI.

**FIGURE 5:**
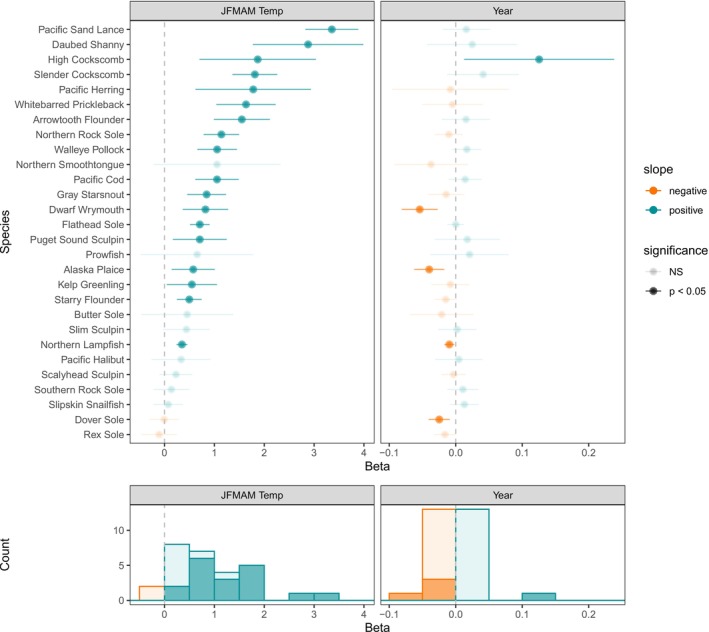
For GOA species, top panels show estimated effect sizes (with 95% CIs) from models with JFMAM temperature (left) and models with a linear year effect (right). Histograms summarize effect sizes and significance across GOA species in the lower panels.

**FIGURE 6:**
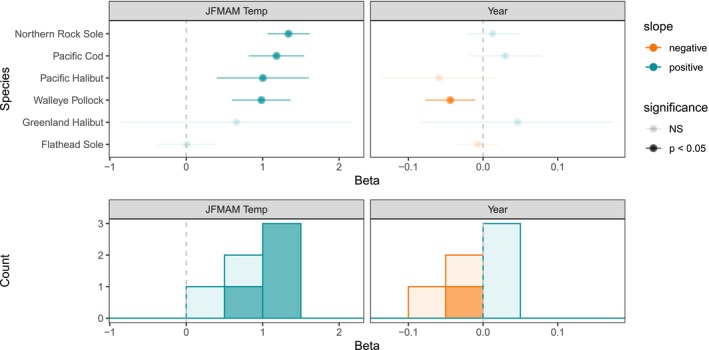
For EBS species, top panels show estimated effect sizes (with 95% CIs) from models with JFMAM temperature (left) and models with a linear year effect (right). Histograms summarize effect sizes and significance across EBS species in the lower panels.

Temperature explained changes in larval size‐at‐date both in terms of the common trend among species and for many single‐species examinations (Figures 3, 4, 5, 6). In both regions, the shared trend in size‐at‐date was significantly correlated with winter–spring (JFMAM) temperatures, with larger size‐at‐date in warmer years. Thermal conditions explained 55% and 64% of interannual variation in the shared size trend for the GOA and EBS, respectively. In the EBS, the relationship was tightest at colder temperatures, indicating a strong association between delayed phenology and cool temperatures (Figure 3). For single species models with temperature covariates, mean JFMAM temperature was a significant predictor of larval size‐at‐date for 18 of 28 GOA species (64%) and 4 of 6 EBS species (67%) (Figures 4, 5, 6). In all cases of statistical significance, larval size‐at‐date was greater in warmer years. Looking across all estimated effects, regardless of significance, 26 of 28 GOA species had positive estimated effects of temperature on size, and only 2 had negative estimated effects (Figure 5). In the Bering Sea, positive effects of temperature on size were observed in all six species (Figure 6). For species present in both systems, the sign of response agreed across systems, although the significance differed for Pacific halibut (non‐significant in the GOA) and flathead sole (non‐significant in the EBS). A simulation for the data‐rich GOA walleye pollock showed that temperature effects on larval size were estimable with as few as 10 observations per year, supporting our chosen sample‐size cutoff (Figure S4).

For most species, we did not find evidence of linear trends in larval length through time. Estimated year trends were non‐significant for 23 out of 28 species in the GOA, and 5 of 6 species in the EBS (Figures 5, 6). In the GOA, a significant positive trend was found for high cockscomb, and significant negative trends were found for dwarf wrymouth, Dover sole, Alaska plaice, and northern lampfish. In the EBS, a significant negative long‐term trend was found for walleye pollock.

Temperature trends in each region were not significantly different from zero for the period of study (all *p* > 0.1; Figure S5). That is, when considering JFMAM temperatures during the period 1972–2022, as well as for only the years for which we had larval size data in each system, neither system showed evidence of a warming (or cooling) trend.

Overall, spatiotemporal model results were robust to the choice of model family, with similar distributions of positive versus negative temperature effects and linear trends across gaussian, lognormal, and generalized gamma models (Figures S6–S9).

## 4 Discussion

We found evidence for widespread thermal sensitivity in the phenology of early life stages of fishes in the Gulf of Alaska and Bering Sea, with a majority of species advancing their phenology in warmer years as exhibited by the larger size obtained by larvae on a given date. The thermal sensitivity of larval phenology was nearly universal across taxa in the GOA and EBS. However, few species showed long‐term trends in size‐at‐date, likely reflecting the lack of a linear trend in ocean temperatures in these regions over the years included in this study. Despite variable survey coverage and timing, the use of spatiotemporal models to estimate changes in larval size‐at‐date allowed us to draw inferences within and across 29 species of fishes in the Gulf of Alaska and Bering Sea combined, resulting in the most comprehensive analysis of changing fish phenology in these systems to date.

Nearly all species investigated showed evidence of larger size‐at‐date in warmer years. Sensitivity of species phenology to thermal conditions is widespread, though not universal, in marine ecosystems (Poloczanska et al. 2013; Thackeray et al. 2010), likely reflecting the underlying thermal‐dependence of physiological rates. However, the exact mechanisms linking temperature and larval size in our study systems are unknown and likely vary by species, and possibly over time. Reproductive timing is temperature‐dependent for many species (Alix et al. 2020), and both walleye pollock and Pacific cod have been shown to advance their timing of spawning in warmer years (Rogers and Dougherty 2019; Miller et al. 2024; Almeida et al. 2024), leading to older and larger larvae on a given date. The development timing of eggs is also linked to temperature, and can vary at least on the order of a week under the range of thermal conditions in this study (IPHC 1998; Blood et al. 1994; Laurel and Blood 2011), resulting in earlier hatching with warmer conditions. Post‐hatching, laboratory studies find strong evidence for temperature‐dependent larval growth in the lab (Laurel et al. 2008, 2016), although field data show mixed evidence of this, possibly due to variation in prey resources or poor fitness of slow growers. For the two species in this study (walleye pollock and Pacific cod) where larval ages are known based on daily otolith increments, variation in larval size was driven by hatch date (age) rather than growth rate, which stayed relatively constant across thermal regimes (Dougherty et al. 2018; Rogers and Dougherty 2019; Miller et al. 2024). However, the relative importance of temperature effects on age versus growth rates and their impact on larval size likely varies among species. Even within a species, as the time since hatching increases, variation in growth is likely to become a relatively more important determinant of size than hatch date.

Additional explanations for the link between temperature and size‐at‐date could be related to hatch size, mortality, or spatial shifts. Variation in size‐at‐hatch has been linked to parental size or condition (maternal effects) and environmental conditions, and could potentially be associated with larval size‐at‐date (Fennie et al. 2024; Pepin et al. 1997; Chambers and Leggett 1996). However, size‐at‐hatch tends to decrease with warming particularly for cold‐water species (Chambers 1997), which would lead to smaller, not larger, size‐at‐date in warmer years. Changes in mortality rates can also affect the mean size of larvae, with higher mortality generally resulting in a population with younger (and smaller) larvae at a given time (Houde 1987). It is unclear to what extent temperature‐driven changes in mortality could contribute to our results, or whether such a mechanism would be shared across species. Finally, observed changes in larval size‐at‐date could reflect changes in the proportional contribution of different spawning groups or overall changes in spawning location within a region. For instance, the spawning of walleye pollock in the Bering Sea varies latitudinally such that aggregations in northerly locations spawn later than those in southerly locations due to colder temperatures and delayed maturation (Bacheler et al. 2012). These spatiotemporal spawning dynamics can affect larval size as the proportional contribution of larvae originating from different locations may vary year to year. Similarly, in the Barents Sea, variation in size of age‐0 Atlantic cod has been associated with north/south shifts in spawning location, reflecting latitudinal differences in spawn timing, growth conditions, and/or size‐at‐hatch (Langangen et al. 2025).

Our study included a diverse range of species life histories and spawning strategies, ranging from species like arrowtooth flounder that spawn in deep waters on the continental slope in winter (Blood et al. 2007), to nearshore spawners like Pacific herring, which spawn in shallow, intertidal, and subtidal vegetated habitats in the spring (Haegele and Schweigert 1985). Some species are short‐lived (e.g., the lifespan of Pacific sand lance ranges from 3 to 7 years; Robards et al. 1999), while others are long‐lived (e.g., Pacific halibut have been found to live up to 55 years; IPHC 1998). Additionally, some species spawn single batches of eggs over a short period of time (e.g., Pacific cod; Stark 2007), while others spawn multiple batches over weeks or months (e.g., pollock, Hinckley 1990; northern smoothtongue, Mason and Phillips 1985), resulting in a prolonged supply of larval cohorts in the plankton. Given the differences in habitats and spawning strategies, it is likely that species differ in their environmental cues, as well as the environment experienced prior to, during, and after spawning and hatching (see Doyle et al. 2019 for an extended life history review). This might explain why some species did not demonstrate thermal sensitivity or share the common size trend across species. In the GOA, rex sole and Dover sole did not exhibit similar patterns in size‐at‐date compared to other species. These flatfishes are both deepwater spawners that migrate to the continental slope to spawn (Abookire and Bailey 2007), where thermal conditions are more stable year‐round than on the shelf. Both species have relatively long pelagic larval durations (8+ months in contrast to 2–4 months for most other species; Table S1), and obtain a relatively large size as larvae. For these species, as well as many others, it is likely that the thermal measure used here (mean Jan–May temperature) was not well matched to their ecology. Finally, for some species, spawn timing has been linked to factors other than (or in addition to) temperature, such as demography (e.g., spawner age in GOA pollock; Rogers and Dougherty 2019). Despite the diverse array of early life history strategies and traits exhibited by the taxa examined in the present study, we found a surprising degree of synchronicity in size‐at‐date, suggesting that thermal conditions are a common driver of phenology across most species in these systems.

Large‐scale studies of phenology in marine, terrestrial, and freshwater systems often find long‐term trends towards earlier spring phenology (Thackeray et al. 2010; Poloczanska et al. 2013). However, we found that the majority of species did not have long‐term (linear) trends in size‐at‐date across the period of study. Most species showed no significant long‐term trends, and a few showed significant negative trends, which would indicate a trend towards smaller size‐at‐date, or later phenology. Although this pattern differs from expectations based on larger‐scale studies, the lack of consistent linear trends in larval fish phenology in the Gulf of Alaska and Bering Sea can be explained by the lack of long‐term winter–spring temperature trends in either system across the time frame of this study. While warming trends have been reported over longer periods, as well as for annual (as opposed to winter–spring) temperatures (Danielson et al. 2020, 2022), those were not evident in mean Jan.‐May temperatures from 1972 to 2022. For both systems, however, this time frame included a wide range of thermal conditions, for example, an extended cold period (2007–2012) and a recent warm period (2014–2019), which provided strong contrast in environmental conditions across which variation in phenology could be investigated. By including temperature directly in our models, as opposed to only testing for long‐term trends, we uncovered a strong relationship between thermal conditions and early life stage phenology across high‐latitude fish species. Given the future temperature increases projected for these regions (Hermann et al. 2019), our results suggest a long‐term phenological shift towards earlier timing may become evident for many species in the future.

The detection of phenological shifts in marine systems is often hindered by the lack of observations at a sufficient temporal resolution, particularly when assessing changes using an occurrence‐based central tendency metric (e.g., date of first occurrence, or mean date of occurrence). For instance, Rademaker et al. (2024) found that the detection of phenological shifts in North Sea herring was only possible with high resolution (daily to weekly) sampling, and not with monthly data. While local, spatially constrained survey efforts may be able to conduct monthly or higher resolution sampling (Greve et al. 2005; Auth et al. 2018; Langan et al. 2021; Rademaker et al. 2024), most larger‐scale ecosystem surveys, such as the CalCOFI survey along the west coast of the United States (Asch 2015) or ichthyoplankton surveys in the North Sea (ICES 2024), sample quarterly or less frequently. To analyze historical survey data collected at a quarterly or lower temporal resolution, previous approaches have grouped data across years (Chen et al. 2025; Walsh et al. 2015; Asch 2015) or across thermal or climate regimes (Smart et al. 2012) to investigate changes in species phenology. However, such grouping of data across years precludes the study of interannual variation in phenology and may mask underlying drivers of changes. Furthermore, changes in the spatial coverage of sampling can bias or mimic phenological changes (de Keyzer et al. 2017), further complicating the inference of phenological shifts from large‐scale marine sampling programs. We found that using a novel phenology metric (i.e., larval size‐at‐date), as well as spatiotemporal models to account for changes in sampling in space and time, allowed us to maximize the use of information from surveys. Although size‐at‐date is not a typical phenology metric measured in units of change in days per year or decade, this metric is indicative of the developmental stage of larval fishes, representing both age (or day of hatching) and progression toward size‐dependent life history transitions such as transformation or settlement.

Long‐term ichthyoplankton sampling in the GOA and EBS, while extensive, was not well designed to monitor species phenology; surveys are often conducted only once per year, with supplemental sampling in some years in certain areas, and survey timing and extent have differed. In the EBS, previous studies using data from many of the same surveys used here have found that spawning for pollock and other species is relatively fixed in time and less variable than spawning location (Bacheler et al. 2012; Vary et al. 2023; Howard et al. 2024; although see Smart et al. 2012). These studies were based on the abundance and distribution of early life stages, including eggs and larvae, but did not use information on size. Inferences were often based on a single or only a few surveys in a year, making it difficult to distinguish temporal shifts due to the confounding with interannual differences in abundance. By analyzing larval size, rather than larval occurrence or abundance, we were able to infer changes in phenology from this long‐term ichthyoplankton dataset for a large number of species, including ones not particularly well sampled by the gear. This approach to studying larval phenology is particularly well suited for high‐latitude or temperate systems because for most species, spawning is constrained in time, resulting in one seasonal pulse of larval production (Doyle et al. 2019). This contracted period of reproduction results in a predictable increase in mean larval size through the season, as larvae recruit to the sampling gear, grow, and then outgrow the sampling gear; this increase is estimated as a smooth term within the spatiotemporal models. However, in lower‐latitude systems, where protracted spawning over months or across seasons is more common, we might not expect to see a continuous increase in mean larval size throughout the season due to the continuous recruitment of newly‐hatched larvae and the ageing out of the larger larvae (Auth et al. 2018). Hence, the suitability of using larval size as a phenology metric can depend on species spawning strategies and their temporal overlap with sampling. However, it is likely that many sampling programs beyond those in the current study could benefit from applying this approach, particularly in higher latitude systems.

The ecological consequences of variation in, and thermal sensitivity of, fish phenology in these systems are unknown, and the future consequences under projected climate change will partly depend on the thermal sensitivity of other processes. According to the match‐mismatch hypothesis, early larval survival and eventual recruitment success depend on the synchronization of first‐feeding larvae with production of their zooplankton prey, which is stimulated by the onset of the spring phytoplankton bloom (Cushing 1990; Platt et al. 2003). An important question then is to what extent spring bloom timing varies with temperature. Modeling work by Asch et al. (2019) suggests that stratification, as well as temperature, is important for bloom timing in higher latitude systems, which can cause fish larvae to become desynchronized from their prey under future warming scenarios. Indeed, in the Bering Sea, the timing of the spring phytoplankton bloom is closely tied to the timing of sea‐ice breakup, but in years with early ice breakup or no ice, the bloom can be delayed by spring storms that delay the onset of stratification (Nielsen et al. 2023). The lack of a consistent temperature‐bloom timing relationship in the Bering Sea may suggest a greater potential for mismatch dynamics if the timing of spawning and larval first feeding becomes earlier with projected warming, particularly in ice‐free years when phytoplankton bloom timing may be delayed. However, in contrast to spring bloom timing, zooplankton production timing does appear to be thermally dependent (Sullaway et al. 2025), suggesting that fish larvae may remain synchronized with their prey in warm and cool conditions. In the Gulf of Alaska, the timing of the phytoplankton bloom has not been clearly tied to thermal conditions, and in recent heatwave years the spring bloom was anomalously delayed (Gann et al. 2022), pointing to potential mismatch if most fish spawn earlier in warmer years. However, some species may be better adapted than others to withstand periods of “mismatch” due to, for example, broader spawning extents, prolonged spawning durations, or higher resilience to starvation (Laurel et al. 2021). Spatial dynamics may also play a role, as some species show more flexibility to shift spawning locations to track optimal habitat (Vary et al. 2023). In the Norwegian Sea, phytoplankton bloom timing has been delayed by coastal water darkening, yet Atlantic cod spawn timing has remained synchronized with bloom timing, suggesting some species may use additional cues beyond temperature to match reproductive timing to prey production, thereby overcoming the risk of increasing trophic asynchrony with climate warming (Opdal, Lindemann, et al. 2024; Opdal, Wright, et al. 2024).

Beyond match‐mismatch dynamics during the first week post‐hatch, variation in larval phenology can be important for processes throughout the first year of life for fishes. For any particular species, changes in larval size‐at‐date (size in late May in this study) are likely to translate into changes in size at the end of the first summer, affecting which prey age‐0 juveniles are able to ingest, and possibly altering their energy reserves going into the first winter. Slight changes in timing and size can have more complex consequences when considering multi‐species interactions. In a two‐species system, Borcherding et al. (2010) found that small changes in hatching phenology can lead to a switch from competitive to predator–prey interactions by the first summer, thereby altering species interactions in a way that affects survival and growth for both species. Beyond the first year of life, increases in larval size‐at‐date associated with future warming could carry over to affect size‐at‐age for older life stages, possibly contributing to decreases in age‐at‐maturity and asymptotic size, which have been observed for many species associated with warming (Neuheimer and Grønkjær 2012; Baudron et al. 2014; Pilipaitytė et al. 2025); however, the extent to which phenology could affect life‐history characteristics beyond the first year requires further research. Although we do not know the consequences of the changes in phenology we inferred in fishes from the Bering Sea and Gulf of Alaska, it is likely that there will be complex changes that are hard to predict, but important to track, as the environment continues to change.

While the ecological consequences will require further study to disentangle, changes in spawn timing and larval phenology can have practical implications for fisheries resource monitoring and assessment, particularly as they may affect the availability of spawning adults or larval recruits to surveys designed to monitor their abundance (Olmos et al. 2023; Rogers et al. 2025). Changes in spawn timing can further create challenges for the management and execution of fisheries that target spawning aggregations, which may move not only in space but also in time. While the need to adapt fisheries management to climate‐driven changes in species geographic distributions has been increasingly acknowledged, changes in phenology remain relatively understudied, particularly in fishes, and thus approaches to adapt to such changes are nascent. Detecting and understanding changes in phenology are the first steps towards developing scientific advice and management strategies that are robust to those changes (Karp et al. 2019).

## Author Contributions

**Lauren A. Rogers:** conceptualization, formal analysis, methodology, visualization, writing – original draft, writing – review and editing. **Kelia E. Axler:** investigation, methodology, visualization, writing – review and editing. **Jennifer S. Bigman:** methodology, writing – review and editing.

## Conflicts of Interest

The authors declare no conflicts of interest.

## Supporting information


**Data S1:** gcb70708‐sup‐0001‐Supinfo.pdf.

## Data Availability

The code that supports the findings of this study is openly available at https://github.com/larogers123/larval_length_phenology_gcb and archived on Zenodo at https://doi.org/10.5281/zenodo.18112120. The data that support the findings of this study are openly available on Dryad at https://doi.org/10.5061/dryad.2280gb66b.
