# Supplementary material for: Widespread Phenological Shifts With Temperature in Alaska's Marine Fishes
Source: Glob Chang Biol. 2026 Jan 16;32(1):e70708. doi: 10.1111/gcb.70708 (PMC12809875; doi:10.1111/gcb.70708)
Supplement: Supplementary file 1 — Data S1: gcb70708‐sup‐0001‐Supinfo.pdf. [file GCB-32-e70708-s001.pdf]

The following supporting information accompanies the article

Widespread phenological shifts with temperature in Alaska's marine fishes

Lauren A. Rogers*, Kelia E. Axler, Jennifer S. Bigman

Global Change Biology

*Corresponding author: lauren.rogers@noaa.gov

Supporting information

Table S1: Life history traits relevant to early life ecology of Gulf of Alaska and Bering Sea fish species included in the study.

Figure S1: Map of sampling events colored by day of year for all surveys included in this study.

Figure S2: Approximate spawning period and pelagic larval duration of species included in this study.

Figure S3: Comparison of estimated DFA trends in larval length-at-date for GOA species with incrementally stricter criteria for time series inclusion.

Figure S4: Simulation results for GOA walleye pollock testing for detection of a temperature effect with small sample sizes.

Figure S5: Interannual variation and time trends in average Jan-May temperatures for the Gulf of Alaska and Bering Sea.

Figure S6: Results from spatiotemporal models fit using a generalized gamma error distribution for Gulf of Alaska species.

Figure S7: Results from spatiotemporal models fit using a lognormal error distribution for Gulf of Alaska species.

Figure S8: Results from spatiotemporal models fit using a generalized gamma error distribution for Bering Sea species.

Figure S9: Results from spatiotemporal models fit using a lognormal error distribution for Bering Sea species.

Figures S10-S13: Residual quantile-quantile plots.

Supplemental References

Table S1: Life history traits relevant to early life ecology of Gulf of Alaska (n = 28) and Bering Sea (n = 6) fish species included in the study. Life history and ecological information were compiled from Doyle et al. (2019; Table 1), Marshall et al. (2019; Table 3), Matarese et al. (1989, 2003), as well as NOAA AFSC's Life History Database (https://apps-afsc.fisheries.noaa.gov/refm/reem/lhweb/index.php), Maximum Age Database (https://apps-afsc.fisheries.noaa.gov/refm/age/stats/max_age.htm), and Ichthyoplankton Information System (https://apps-afsc.fisheries.noaa.gov/ichthyo/index.php). Literature references describing spawn timing and pelagic larval duration were cross-referenced and modified based on in situ observations of egg and larval presence from EcoFOCI ichthyoplankton time-series by species and region. Additional sources are indicated.

**Species**	**Common name**	**Longevity (years)**	**Age at First Maturity (years)**	**Fecundity (1000 eggs /female)**	**Spawning Habitat**	**Egg Type**	**Spawning Season**	**Spawn Timing**	**Larval Phase**	**Pelagic Larval Duration (months)**	**Additional Sources** ** aside from Doyle et al. 2019, Marshall et al. 2019, Matarese et al. (1989; 2003), & NOAA AFSC's Life History Table, Max Age Table, IIS, & survey data*
**Gulf of Alaska**											
Ammodytes personatus	Pacific Sand Lance	3-7	1	1–16	Nearshore-Shelf	Demersal, adhesive	Summer-Winter	Sep-Mar	Feb-Jul	8	Spawning - Matta and Baker 2020; Lifespan - Robards et al. 1999, Rogers et al. 1979, Kendall et al. 1980
Anoplarchus insignis	Slender Cockscomb	Unk	Unk	Unk	Nearshore	Demersal, adhesive	Winter-Spring	Jan-Apr	Apr-Jun	3	Matarese et al. 2013
Anoplarchus purpurescens	High Cockscomb	Unk	2-3	2-3	Nearshore	Demersal, adhesive	Winter-Spring	Jan-Mar	Apr-Jun	3	Matarese et al. 2013; Fecundity - Peppar 1965
Artedius harringtoni	Scalyhead Sculpin	Unk	Unk	Unk	Nearshore-Shelf	Demersal, adhesive	Summer-Spring	Dec-Apr	Apr-Dec	Unk	Spawning - Kendall and Dunn 1985, Ragland and Fischer 1987
Atheresthes stomias	Arrowtooth Flounder	37	3-8	130–2240	Deepwater	Deep Pelagic	Fall-Winter	Dec-Feb	Jan-Jul	5	Spawning - Blood et al. 2007; Maximum Age - Turnock et al. 1999
Bathyagonus alascanus	Gray Starsnout	Unk	Unk	Unk	Unk	Demersal, adhesive	Winter-Spring	Mar-Jun	Apr-Jul	3	
Clupea pallasii	Pacific Herring	19	2–3	12–80	Nearshore	Demersal, adhesive	Winter-Spring	Mar-Jun	Apr-Jul	3	Spawning - McGowan et al. 2021; Age - Morrow 1980
Cryptacanthodes aleutensis	Dwarf Wrymouth	Unk	Unk	Unk	Shelf	Demersal	Winter-Spring	Jan-Jun	Mar-Jun	3	Spawning - Pietsch and Orr 2019
Gadus chalcogrammus	Walleye Pollock	31	3	95–1080	Shelf	Deep Pelagic	Winter-Spring	Jan-Jun	Mar-Jul	4	
Gadus macrocephalus	Pacific Cod	17	2	1000–5000	Shelf	Demersal	Winter-Spring	Jan-May	Apr-Jul	3	

Glyptocephalus zachirus	Rex Sole	49	3	4–238	Deepwater	Epipelagic	Fall-Summer	Oct-Aug	Apr-Oct	12	Spawning - Hosie and Horton 1977, Abookire 2006
Hexagrammos decagrammus	Kelp Greenling	18	3	5–900	Nearshore-Shelf	Demersal, adhesive	Fall-Winter	Oct-Mar	Feb-Nov	8	Spawning - ODFW
Hippoglossoides elassodon	Flathead Sole	37	2	50–160	Shelf	Epipelagic	Winter-Summer	Mar-Jul	Apr-Oct	4	Spawning - Hirschberger and Smith 1983; Larval phase - Porter 2005
Hippoglossus stenolepis	Pacific Halibut	55	4	500–4000	Deepwater	Deep Pelagic	Fall-Winter	Nov-Mar (peak: Dec-Jan)	Jan-Aug	5	Spawning - St. Pierre 1984, Fish et al. 2022
Isopsetta isolepis	Butter Sole	11	3	350–650	Nearshore-Shelf	Epipelagic	Winter-Spring	Feb-Apr	Apr-Sep	2	Spawning - Richardson et al. 1980
Lepidopsetta bilineata	Southern Rock Sole	28	3	130–400	Nearshore-Shelf	Demersal, adhesive	Winter-Summer	Feb-Jul (peak: Jun-Jul)	Apr-Oct	4	Spawning - Stark and Somerton 2002, Stark 2004, Porter and Ciannelli 2018
Lepidopsetta polyxystra	Northern Rock Sole	37	3	152–404	Nearshore-Shelf	Demersal, adhesive	Winter-Spring	Jan-Jun (peak: Mar-Apr)	Apr-Oct	5	Spawning - Stark and Somerton 2002, Matta and Anderl 2012
Leptoclinus maculatus	Daubed Shanny	14	6	1-3	Nearshore-Shelf	Demersal	Winter	Jan-Mar	Mar-Oct	24	Age at First Maturity - Meyer Ottesen et al. 2011; Fecundity - Andriyashev 1954, Pethon 2005; Longevity - Meyer Ottesen et al. 2014
Leuroglossus schmidti	Northern Smoothtongue	6	2	5–8	Deepwater	Epipelagic	Winter-Fall	Jan-Nov	Feb-Nov	3	
Liparis fucensis	Slipskin Snailfish	Unk	Unk	1-5	Shelf	Demersal, adhesive	Winter-Summer	Jan-Aug (peak: Jun)	Apr-Sep	2	Fecundity - DeMartini 1978, Spawning - Pietsch and Orr 2019
Microstomus pacificus	Dover Sole	64	5	40–167	Deepwater	Epipelagic	Winter-Summer	Jan-Aug	Apr-Sep	8-24	
Platichthys stellatus	Starry Flounder	21	5	900–3671	Nearshore	Epipelagic	Winter-Summer	Feb-Jul	Mar-Sep	2	
Pleuronectes quadrituberculatus	Alaska Plaice	38	4	56–520	Nearshore-Shelf	Epipelagic	Winter-Spring	Mar-Jun	Apr-Sep	2	Spawning - Bakkala 1993
Poroclinus rothrocki	Whitebarred Prickleback	Unk	Unk	Unk	Shelf	Demersal	Winter-Spring	Feb-Jun	Apr-Jul	4	
Radulinus asprellus	Slim Sculpin	Unk	Unk	Unk	Shelf	Demersal	Winter-Spring	Mar-Jun	Apr-Sep	Unk	
Ruscarius meanyi	Puget Sound Sculpin	Unk	Unk	Unk	Nearshore-Shelf	Demersal	Spring	Mar-Jun	Apr-Sep	3	

Stenobrachius leucopsarus	Northern Lampfish	8	4	12.6	Deepwater	Deep Pelagic	Fall-Spring	Dec-Mar	Feb-Nov	8	Fecundity - King and McFarlane 2003; Spawning - Smoker and Pearcy 1970
Zaprora silenus	Prowfish	20	4	Unk	Deepwater	Demersal	Winter-Spring	Jan-Jun	Mar-Jul	4	Spawning - Pietsch and Orr 2019, Smith et al. 2004
**Bering Sea**											
Gadus chalcogrammus	Walleye Pollock	31	3	95–1080	Shelf	Deep Pelagic	Winter-Summer	Feb-Aug	Mar-Sep	4	Spawning - Hughes and Hirschhorn 1979, Serobaba 1968
Gadus macrocephalus	Pacific Cod	17	2	1000–5000	Shelf	Demersal	Winter-Spring	Feb-Apr (peak: Mar)	Apr-Jul	3	Spawning - Neidetcher et al. 2014
Hippoglossoides elassodon	Flathead Sole	37	2	50–160	Shelf	Epipelagic	Spring-Summer	Apr-Jul (peak: May)	May-Sep	4	Spawning - Stark and Somerton 2002, Stark 2004, Porter and Ciannelli 2018
Hippoglossus stenolepis	Pacific Halibut	55	4	500–4000	Deepwater	Deep Pelagic	Fall-Winter	Nov-Mar (peak: Dec-Jan)	Jan-Aug	5	Spawning - St-Pierre 1984
Lepidopsetta polyxystra	Northern Rock Sole	37	3	152–404	Nearshore-Shelf	Demersal, adhesive	Winter-Spring	Jan-Jun (peak: Mar-Apr)	Apr-Oct	5	Spawning - Stark and Somerton 2002, Doyle et al. 2009, Laurel et al. 2014
Reinhardtius hippoglossoides	Greenland Halibut	53	7–10	23-136	Deepwater	Pelagic	Summer-Spring	Sep-Mar (peak: Nov-Feb)	Feb-Sep	4	Spawning - Sohn et al. 2010, Duffy-Anderson et al. 2013, Shuntov 1970, Bulatov 1983; Pelagic Larval Duration - Coad and Reist 2018

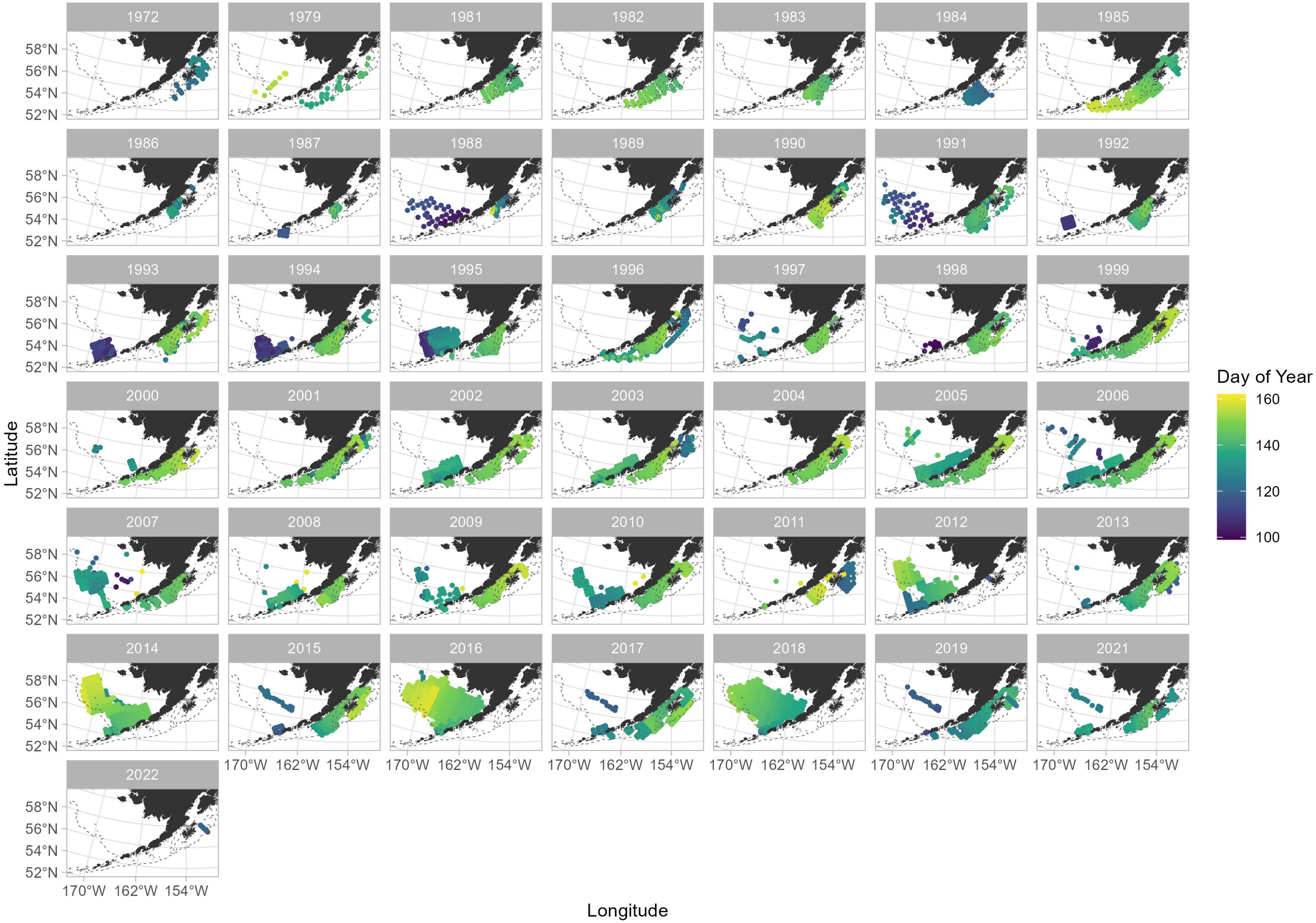

Figure S1: Map of sampling events colored by day of year for all surveys included in this study.

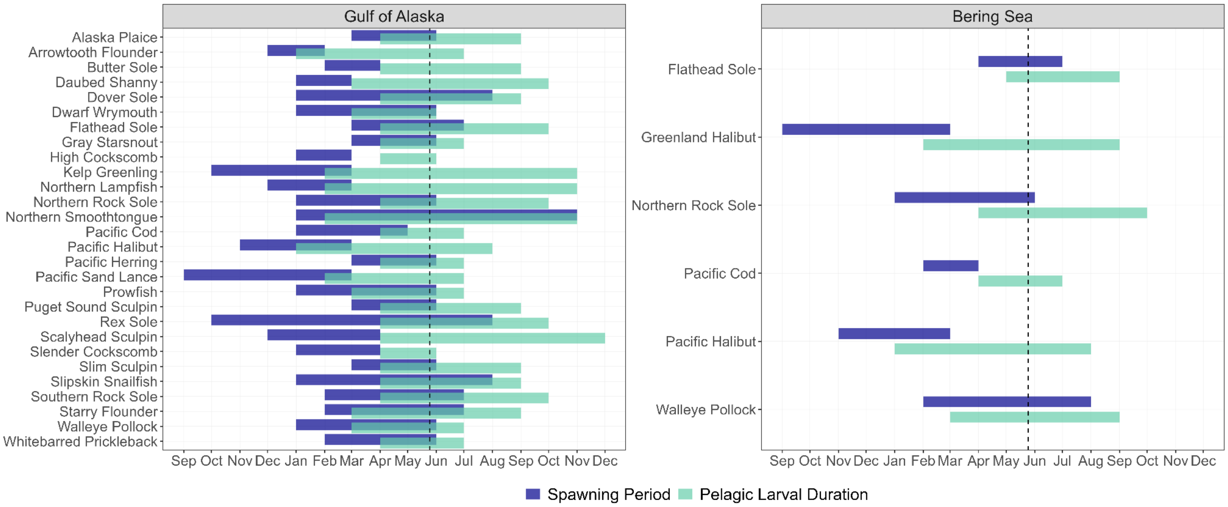

Figure S2: Approximate spawning period and pelagic larval duration of species included in this study. See Table S1 for references.

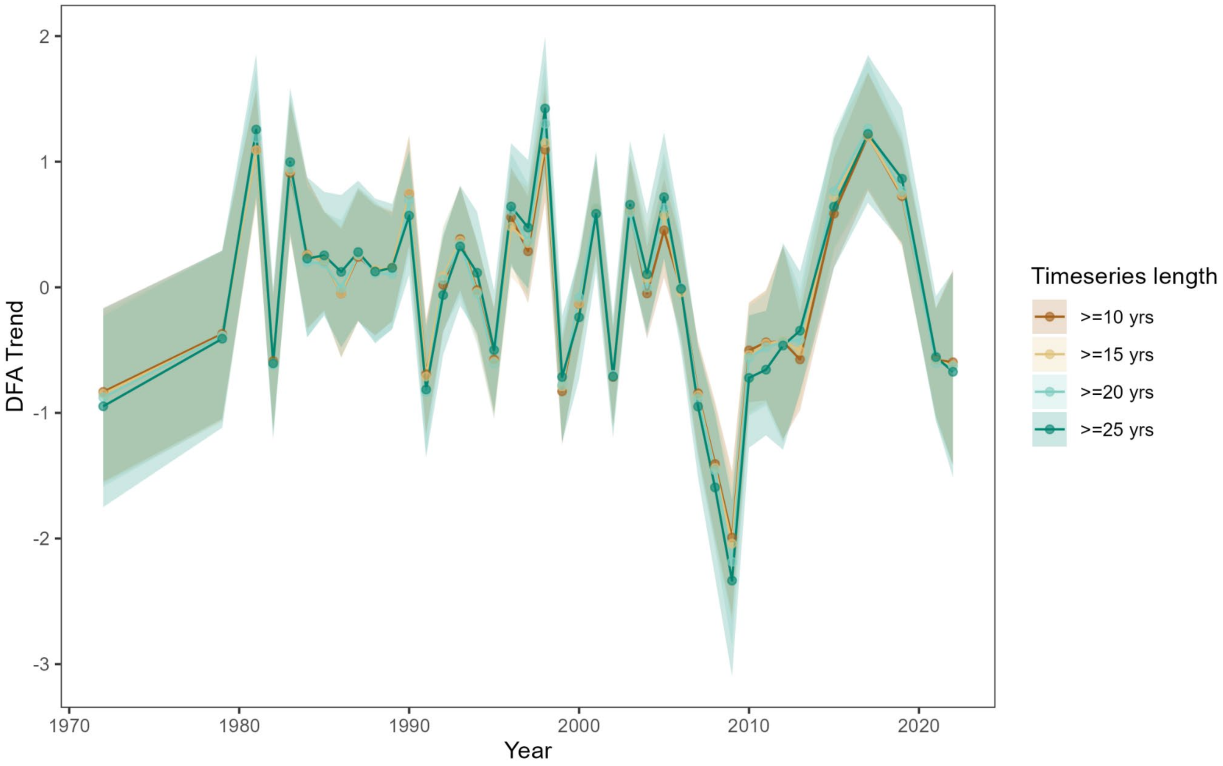

Figure S3: Comparison of estimated DFA trends in larval length-at-date for GOA species with incrementally stricter criteria for time series inclusion. A DFA was conducted using the original full dataset (all species with a minimum of 10 years of at least 10 observations in a year; 28 species), then only species with a minimum of 15 years of observations (n = 24 species), 20 years (n = 18 species), and 25 years (n = 13 species). All estimated trends were highly correlated with one another (all r > 0.98).

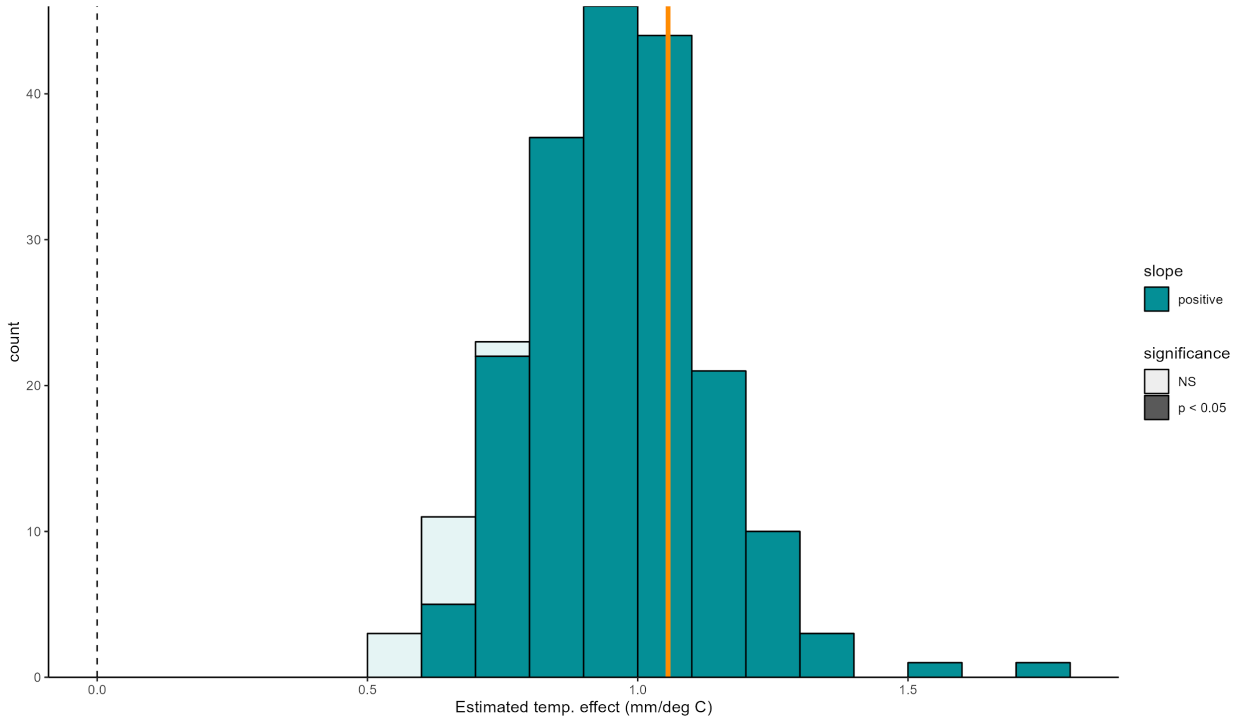

Figure S4: Distribution of estimated temperature effects from spatiotemporal models fit to subsets of larval length observations from GOA walleye pollock. The orange line indicates the estimate from a model fit to all data points (n = 147,303), and histogram shading indicates whether estimates were significantly different from zero ($p < 0.05$). For each of 200 simulations, data were randomly subset to 10 observations per year from the original dataset, for a total of 390 observations per model.

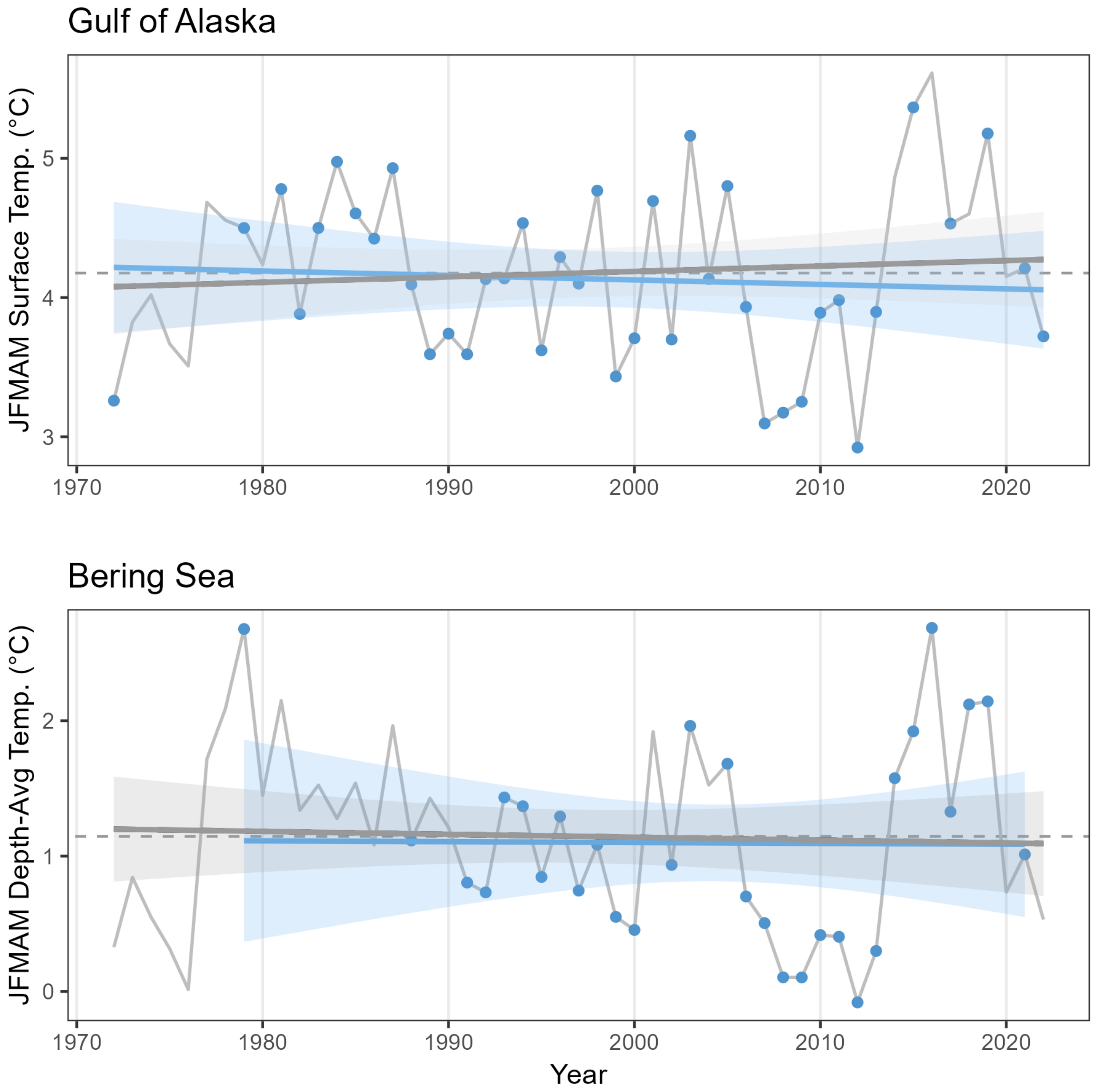

Figure S5: Interannual variation and time trends in average Jan-May temperatures for the (a) Gulf of Alaska and (b) Bering Sea. A linear regression of temperature as a function of year was fitted to all data points (1972-2022; gray lines and shading), as well as to only the blue points (blue lines and shading), which indicate temperatures from the years with larval length measurements available for use in this study. All linear regressions were non-significant ($p > 0.1$).

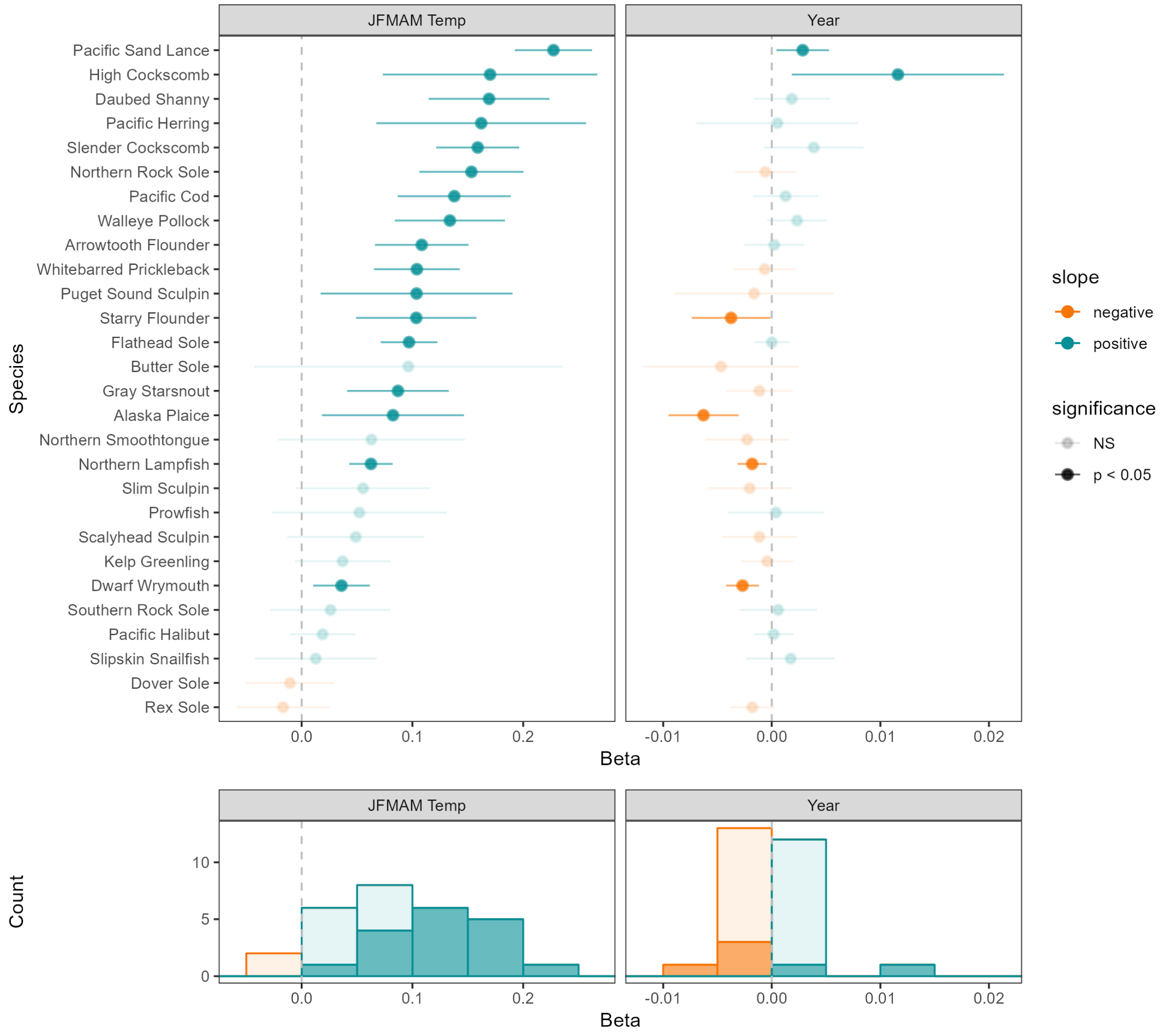

Figure S6: Results from spatiotemporal models fit using a generalized gamma error distribution, for comparison to Figure 5 in the main text. For GOA species, top panels show estimated effect sizes (with 95% CIs) from models with JFMAM temperature (left) and models with a linear year effect (right). Histograms summarize effect sizes and significance across GOA species in the lower panels. A missing point indicates a model that did not converge.

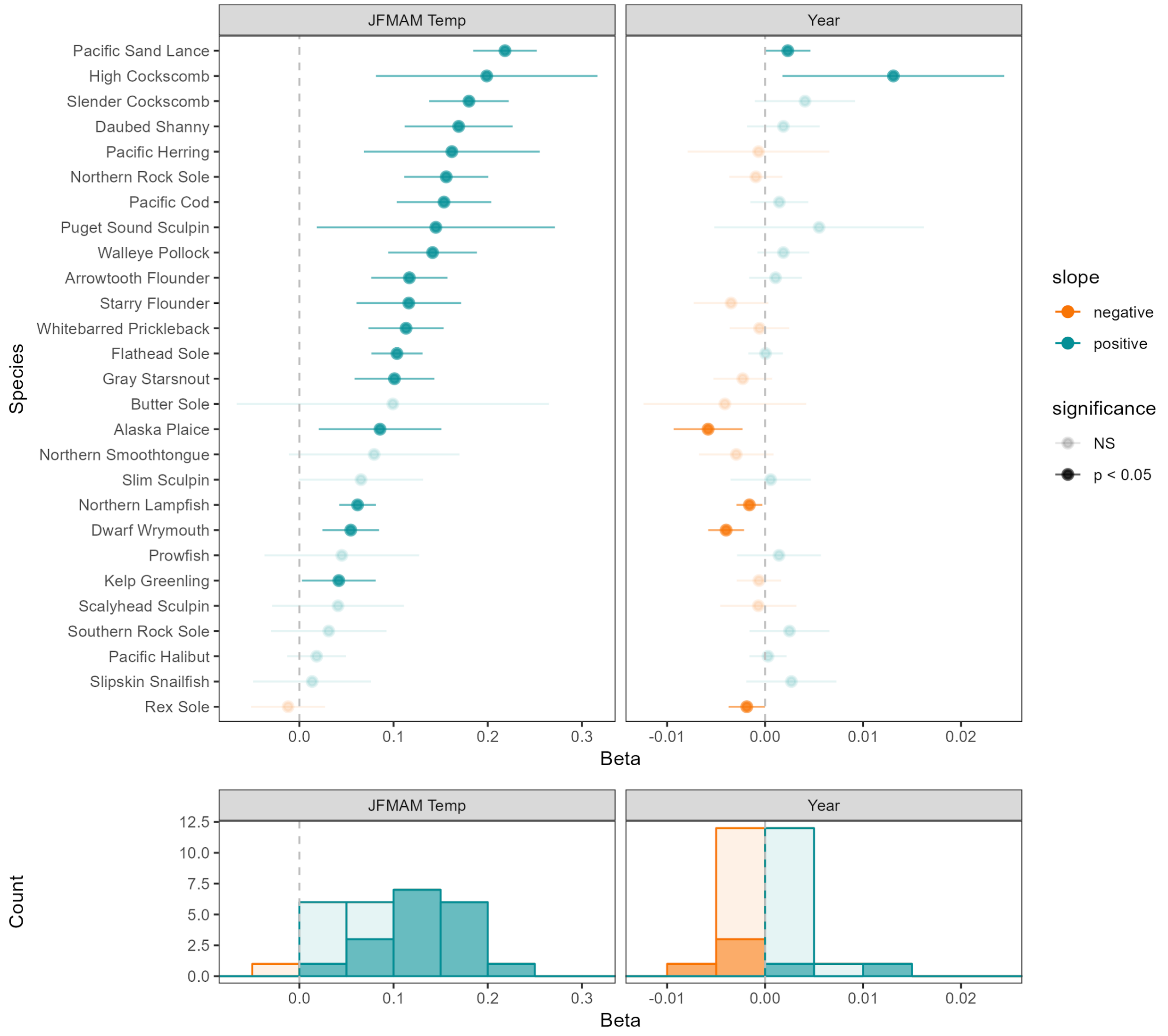

Figure S7: Results from spatiotemporal models fit using a lognormal error distribution, for comparison to Figure 5 in the main text. For GOA species, top panels show estimated effect sizes (with 95% CIs) from models with JFMAM temperature (left) and models with a linear year effect (right). Histograms summarize effect sizes and significance across GOA species in the lower panels. Dover sole is not shown as neither model for this species converged.

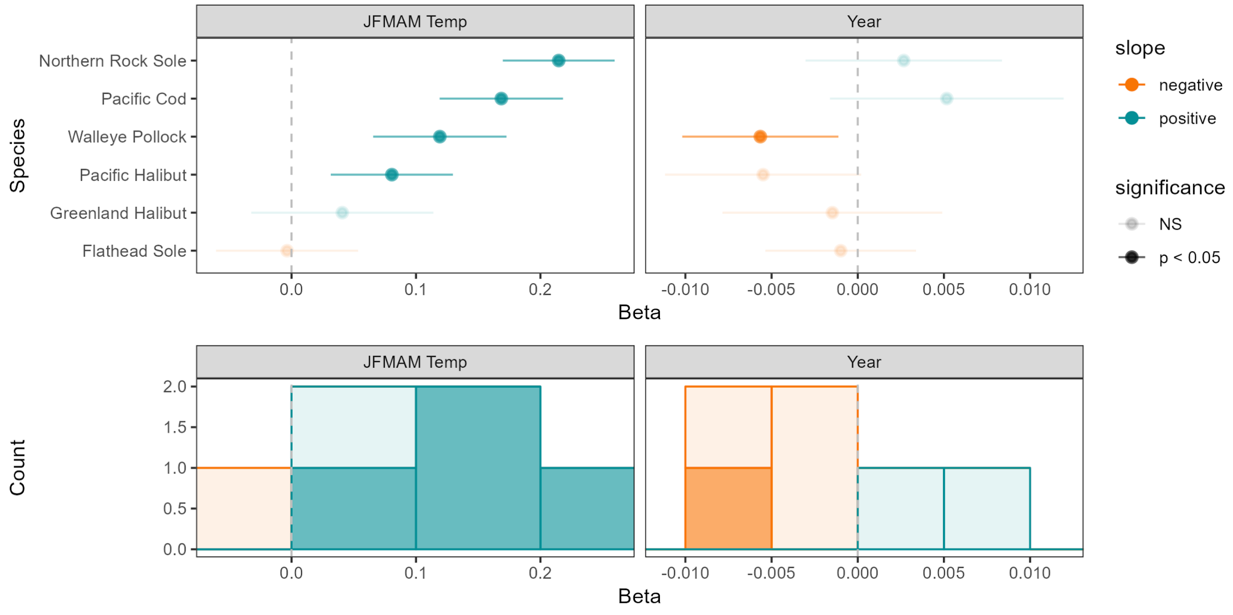

Figure S8. Results from spatiotemporal models fit using a generalized gamma error distribution, for comparison to Figure 6 in the main text. For EBS species, top panels show estimated effect sizes (with 95% CIs) from models with JFMAM temperature (left) and models with a linear year effect (right). Histograms summarize effect sizes and significance across EBS species in the lower panels.

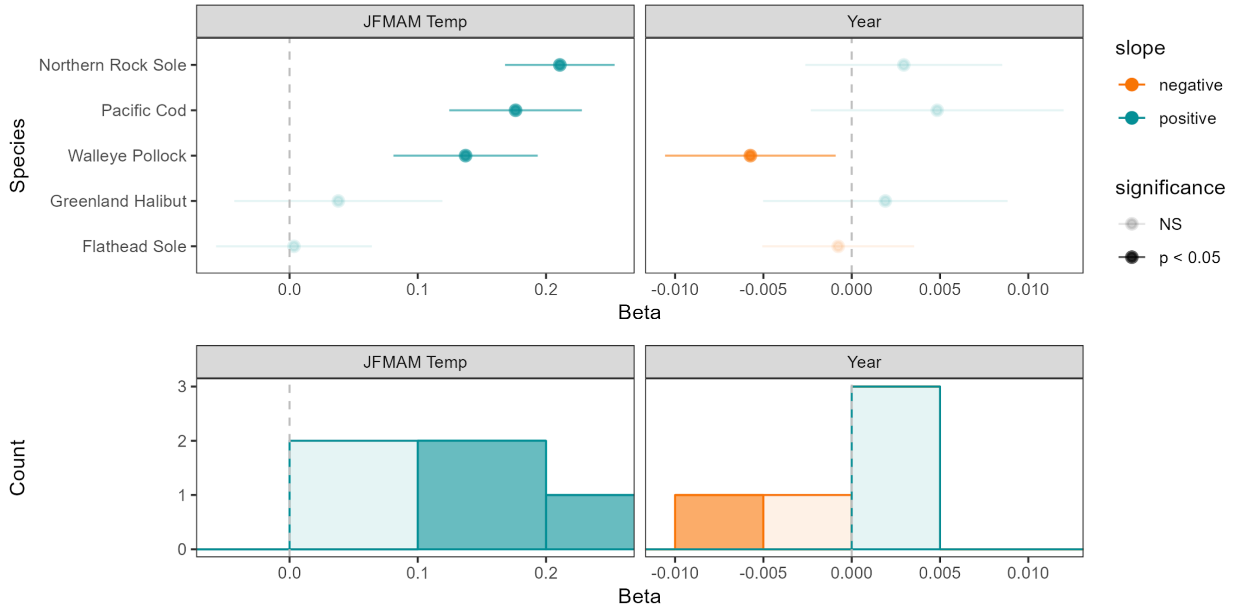

Figure S9. Results from spatiotemporal models fit using a lognormal error distribution, for comparison to Figure 6 in the main text. For EBS species, top panels show estimated effect sizes (with 95% CIs) from models with JFMAM temperature (left) and models with a linear year effect (right). Histograms summarize effect sizes and significance across EBS species in the lower panels. Note that results for Pacific halibut are missing as those models did not converge.

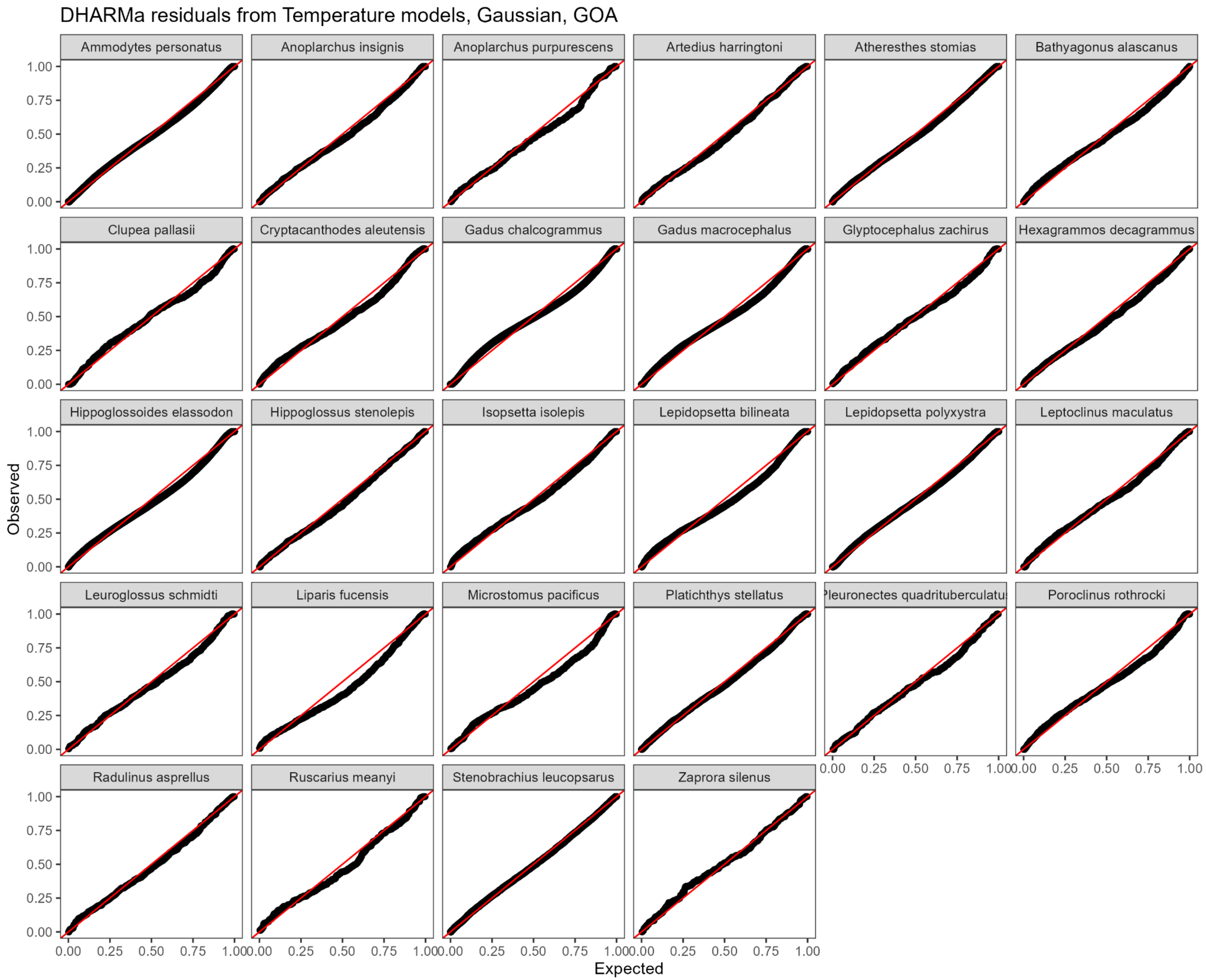
DHARMa residuals from Temperature models, Gaussian, GOA
Ammodytes personatus
Anoplarchus insignis
Anoplarchus purpurescens
Artedius harringtoni
Atheresthes stomias
Bathyagonus alascanus
Clupea pallasii
Cryptacanthodes aleutensis
Gadus chalcogrammus
Gadus macrocephalus
Glyptocephalus zachirus
Hexagrammos decagrammus
Hippoglossoides elassodon
Hippoglossus stenolepis
Isopsetta isolepis
Lepidopsetta bilineata
Lepidopsetta polyxystra
Leptoclinus maculatus
Leuroglossus schmidti
Liparis fucensis
Microstomus pacificus
Platichthys stellatus
Pleuronectes quadrituberculatu
Poroclinus rothrocki
Radulinus asprellus
Ruscarius meanyi
Stenobrachius leucopsarus
Zaprora silenus
Observed
Expected
0.00
0.25
0.50
0.75
1.00

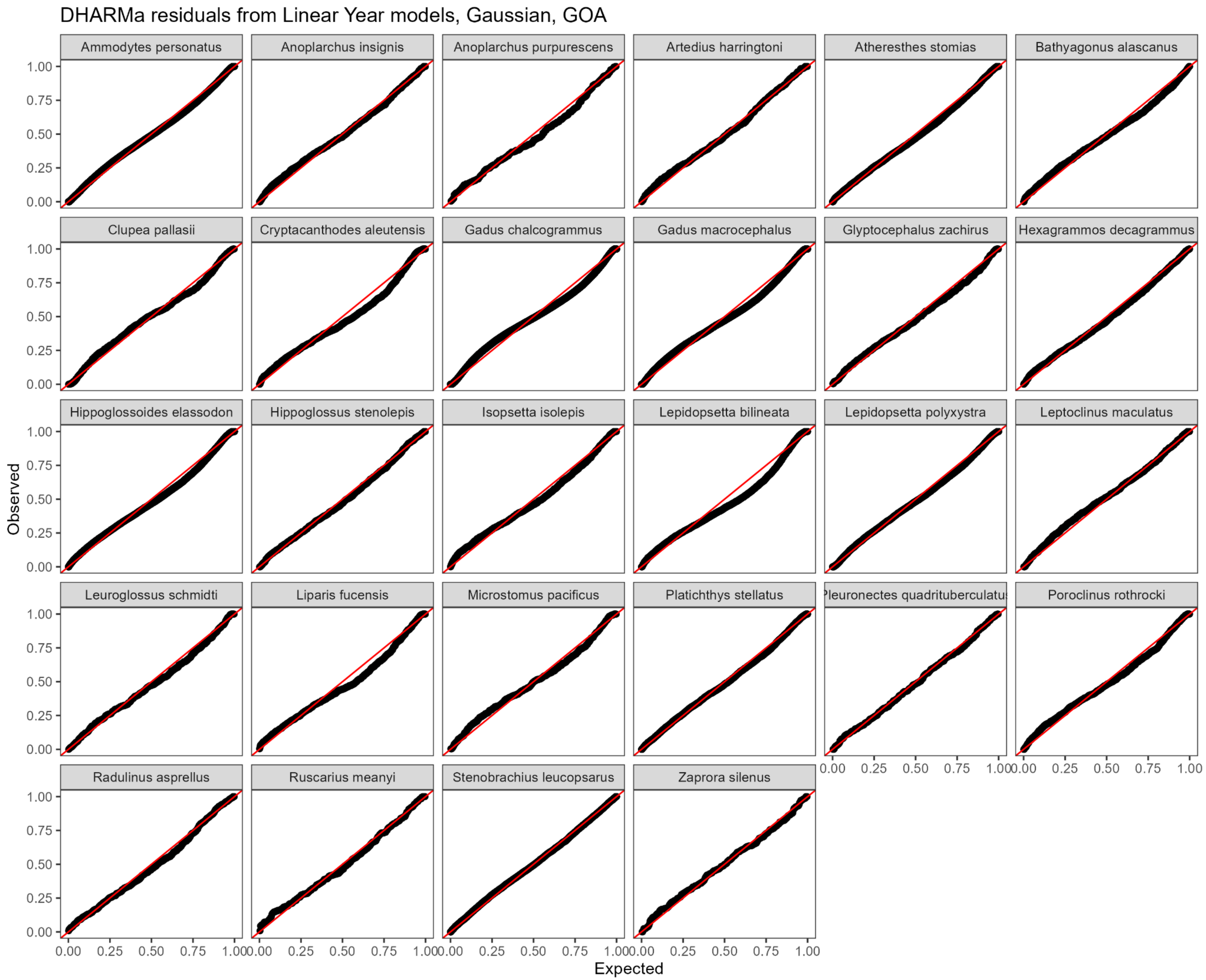

DHARMa residuals from Linear Year models, Gaussian, GOA
Ammodytes personatus
Anoplarchus insignis
Anoplarchus purpurescens
Artedius harringtoni
Atheresthes stomias
Bathyagonus alascanus
Clupea pallasii
Cryptacanthodes aleutensis
Gadus chalcogrammus
Gadus macrocephalus
Glyptocephalus zachirus
Hexagrammos decagrammus
Hippoglossoides elassodon
Hippoglossus stenolepis
Isopsetta isolepis
Lepidopsetta bilineata
Lepidopsetta polyxystra
Leptoclinus maculatus
Leuroglossus schmidti
Liparis fucensis
Microstomus pacificus
Platichthys stellatus
Pleuronectes quadrituberculatus
Poroclinus rothrocki
Radulinus asprellus
Ruscarius meanyi
Stenobrachius leucopsarus
Zaprora silenus
Observed
Expected
0.00
0.25
0.50
0.75
1.00

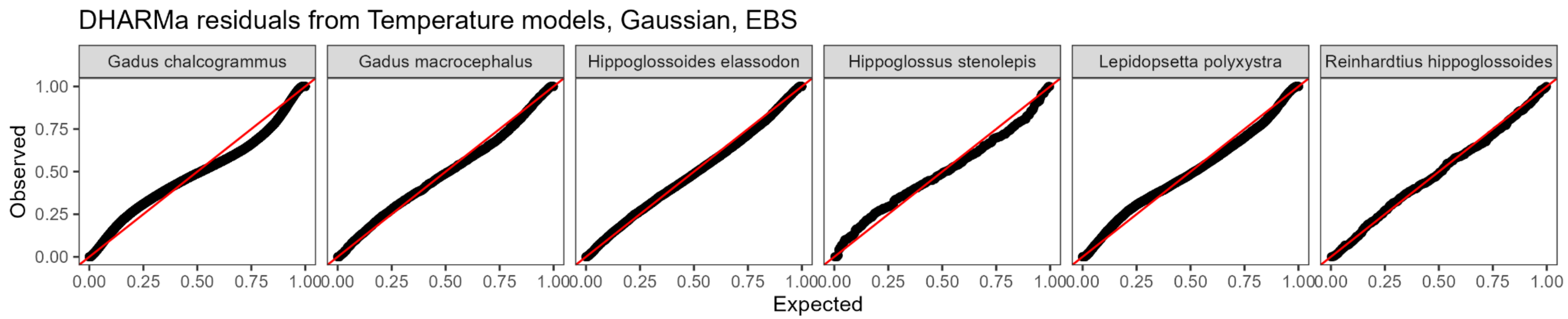

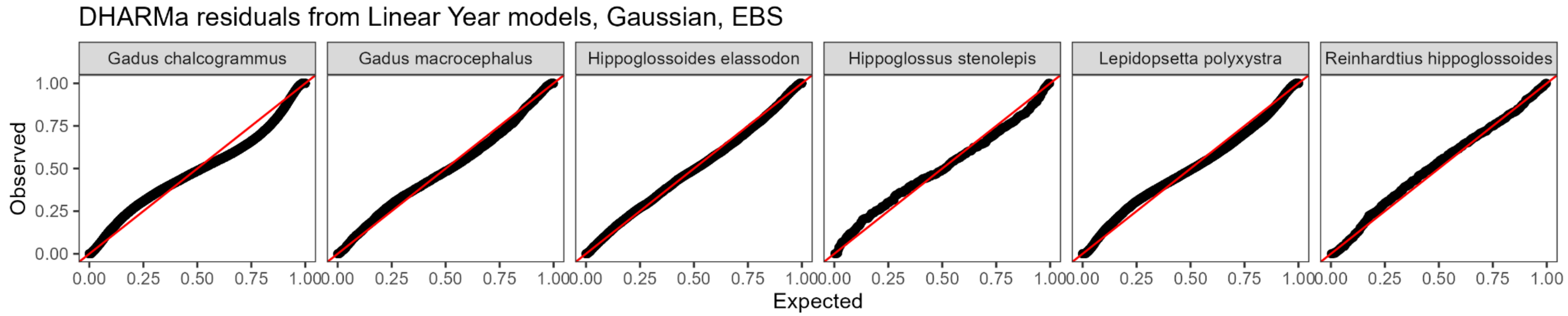

Figures S10-S13: Quantile-quantile plots for spatiotemporal models presented in this study. Shown are simulation-based DHARMa residuals from models with a gaussian response fit to species data from the GOA and EBS, for models with an effect of JFMAM (mean Jan-May) temperature on larval size, and models with an estimated linear trend in size through time (“Linear Year” models).

Supplementary references

Abookire, A. 2006. Reproductive biology, spawning season, and growth of female rex sole (*Glyptocephalus zachirus*) in the Gulf of Alaska. Fishery Bulletin **104**.

Andriyashev, A.P. 1954. Fishes of the northern seas of the U.S.S.R. **53**: 266–269. Zoological Institute of the U.S.S.R. Academy of Sciences. No. 53. pp 266-269.

Bakkala, R.D. 1993. Structure and historical changes in the groundfish complex of the Eastern Bering Sea. NOAA Technical Report NMFS.

Blood, D.M., Matarese, A.C., and Busby, M.S. 2007. Spawning, egg development, and early life history dynamics of Arrowtooth Flounder (*Atheresthes stomias*) in the Gulf of Alaska. U.S. Department of Commerce, National Oceanic and Atmospheric Administration, National Marine Fisheries Service.

Bulatov, D.A. 1983. Distribution of eggs and larvae of Greenland halibut (*Reinhardtius hippoglossoides*) (Pleuronectidae) in the eastern Bering Sea. Journal of Ichthyology **23**: 157–159.

Coad, B.W., and Reist, J.D. 2018. Marine Fishes of Arctic Canada. University of Toronto Press.

DeMartini, E.E. 1978. Apparent paternal care in *Liparis fucensis* (Pisces: Cyclopteridae). Copeia **1978**(3): 537–539. doi:10.2307/1443625.

Doyle, M.J., Picquelle, S.J., Mier, K.L., Spillane, M.C., and Bond, N.A. 2009. Larval fish abundance and physical forcing in the Gulf of Alaska, 1981–2003. Progress in Oceanography **80**(3): 163–187. doi:10.1016/j.pocean.2009.03.002.

Doyle, M.J., Strom, S.L., Coyle, K.O., Hermann, A.J., Ladd, C., Matarese, A.C., Shotwell, S.K., and Hopcroft, R.R. 2019. Early life history phenology among Gulf of Alaska fish species: Strategies, synchronies, and sensitivities. Deep Sea Research Part II: Topical Studies in Oceanography **165**: 41–73. doi:10.1016/j.dsr2.2019.06.005.

Duffy-Anderson, J.T., Blood, D.M., Cheng, W., Ciannelli, L., Matarese, A.C., Sohn, D., Vance, T.C., and Vestfals, C. 2013. Combining field observations and modeling approaches to examine Greenland halibut (*Reinhardtius hippoglossoides*) early life ecology in the southeastern Bering Sea. Journal of Sea Research **75**: 96–109. doi:10.1016/j.seares.2012.06.014.

Fish, T., Wolf, N., Smeltz, T.S., Harris, B.P., and Planas, J.V. 2022. Reproductive biology of female Pacific Halibut (*Hippoglossus stenolepis*) in the Gulf of Alaska. Front. Mar. Sci. **9**. doi:10.3389/fmars.2022.801759.

Hirschberger, W.A. 1983. Spawning of twelve groundfish species in the Alaska and Pacific Coast regions, 1975-81. Northwest and Alaska Fisheries Center, National Marine Fisheries Service, National Oceanic and Atmospheric Administration, Seattle, Wash.

Hosie, M.J., and Horton, H.F. 1977. Biology of the rex sole, *Glyptocephalus zachirus*, in waters off Oregon. Fishery Bulletin **75**: 51–60.

Hughes, S.E., and Hirschhorn, G. 1979. Biology of walleye pollock, *Theragra chalcogramma*, in western Gulf of Alaska, 1973-75. Fishery Bulletin **77**(1): 263–274.

Kendall, A.W., and Dunn, J.R. 1985. Ichthyoplankton of the Continental Shelf Near Kodiak Island, Alaska. NOAA Technical Report NMFS 20.

Kendall, A.W., Dunn, J.R., Wolotira, R.J., Bowerman, J.H., Dey, D.B., Matarese, A.C., and Munk, J.E. 1980. Zooplankton, including ichthyoplankton and decapod larvae, of the Kodiak Shelf. NWAFC Processed Report 80-8. Available from https://repository.library.noaa.gov/view/noaa/19846 [accessed 2 May 2025].

King, J.R., and McFarlane, G.A. 2003. Marine fish life history strategies: applications to fishery management. Fisheries Management and Ecology **10**(4): 249–264. doi:10.1046/j.1365-2400.2003.00359.x.

Laurel, B.J., Danley, C., and Haines, S. 2014. The effects of temperature on growth, development and settlement of northern rock sole larvae (*Lepidopsetta polyxystra*). Fisheries Oceanography **23**(6): 495–505. doi:10.1111/fog.12084.

Marshall, K.N., Duffy-Anderson, J.T., Ward, E.J., Anderson, S.C., Hunsicker, M.E., and Williams, B.C. 2019. Long-term trends in ichthyoplankton assemblage structure, biodiversity, and synchrony in the Gulf of Alaska and their relationships to climate. Progress in Oceanography **170**: 134–145. doi:10.1016/j.pocean.2018.11.002.

Matarese, A.C. 1989. Laboratory guide to early life history stages of Northeast Pacific fishes. NOAA Technical Report, NMFS. Available from https://repository.library.noaa.gov/view/noaa/5915 [accessed 12 March 2025].

Matarese, A., Blood, D., Picquelle, S., and Benson, J. 2003. Atlas of Abundance and Distribution Patterns of Ichthyoplankton from the Northeast Pacific Ocean and Bering Sea Ecosystems Based on Research Conducted by the Alaska Fisheries Science Center (1972–1996). NOAA Professional Papers NMFS 1. 291 pp.

Matarese, A.C., Blood, D.M., and Busby, M.S. 2013. Guide to the Identification of Larval and Early Juvenile Pricklebacks (Perciformes: Zoarcoidei: Stichaeidae) in the Northeastern Pacific Ocean and Bering Sea. U.S. Department of Commerce, National Oceanic and Atmospheric Administration, National Marine Fisheries Service.

Matta, M.E., and Anderl, D.M. 2012. Northern rock sole (*Lepidopsetta polyxystra*). Resource Ecology and Fisheries Management Division, Alaska Fisheries Science Center, NMFS, NOAA, 7600 Sand Point Way NE, Seattle WA 98115. Available from http://www.afsc.noaa.gov/REFM/Age/default.htm.

Matta, M.E., and Baker, M.R. 2020. Age and growth of Pacific sand lance (*Ammodytes personatus*) at the latitudinal extremes of the Gulf of Alaska large marine ecosystem. nwnt **101**(1): 34–49. doi:10.1898/1051-1733-101.1.34.

McGowan, D.W., Branch, T.A., Haught, S., and Scheuerell, M.D. 2021. Multi-decadal shifts in the distribution and timing of Pacific herring (*Clupea pallasii*) spawning in Prince William Sound, Alaska. Can. J. Fish. Aquat. Sci. **78**(11): 1611–1627. doi:10.1139/cjfas-2021-0047.

Meyer Ottesen, C., Hop, H., Christiansen, J., and Falk-Petersen, S. 2011. Early life history of the daubed shanny (Teleostei: *Leptoclinus maculatus*) in Svalbard waters. Marine Biodiversity **41**: 383–394. doi:10.1007/s12526-010-0079-3.

Meyer Ottesen, C.A., Hop, H., Falk-Petersen, S., and Christiansen, J.S. 2014. Growth of daubed shanny (Teleostei: *Leptoclinus maculatus*) in Svalbard waters. Polar Biol **37**(6): 809–815. doi:10.1007/s00300-014-1481-2.

Morrow, J.E. 1980. The Freshwater Fishes of Alaska. Alaska Northwest Publishing Company.

Neidetcher, S.K., Hurst, T.P., Ciannelli, L., and Logerwell, E.A. 2014. Spawning phenology and geography of Aleutian Islands and eastern Bering Sea Pacific cod (*Gadus macrocephalus*). Deep Sea Research Part II: Topical Studies in Oceanography **109**: 204–214. doi:10.1016/j.dsr2.2013.12.006.

Peppar, J.L. 1965. Some features of the life history of the cockscomb prickleback: *Anoplaruchus purpurescens*. University of British Columbia. Available from https://open.library.ubc.ca/soa/cIRcle/collections/ubctheses/831/items/1.0302515 [accessed 30 April 2025].

Pethon, P. 2005. Aschehougs store fiskebok. *In* 5th edition. Aschehoug.

Pietsch, T.W., and Orr, J.W. 2019. Fishes of the Salish Sea. University of Washington Press. Available from https://uwapress.uw.edu/book/9780295743745/fishes-of-the-salish-sea/ [accessed 30 April 2025].

Porter, S.M. 2005. Temporal and spatial distribution and abundance of flathead sole (*Hippoglossoides elassodon*) eggs and larvae in the western Gulf of Alaska. Fishery Bulletin **103**: 648–658.

Porter, S.M., and Ciannelli, L. 2018. Effect of temperature on Flathead Sole (*Hippoglossoides elassodon*) spawning in the southeastern Bering Sea during warm and cold years. Journal of Sea Research **141**: 26–36. doi:10.1016/j.seares.2018.08.003.

Ragland, H.C., and Fischer, E.A. 1987. Internal Fertilization and Male Parental Care in the Scalyhead Sculpin, *Artedius harringtoni*. Copeia **1987**(4): 1059–1062. doi:10.2307/1445578.

Richardson, S.L., Dunn, J.R., and Naplin, N.A. 1980. Eggs and larvae of butter sole, *Isopsetta isolepis* (Pleuronectidae), off Oregon and Washington. Fishery Bulletin **78**(2): 401–417.

Robards, M.D., Piatt, J.F., and Rose, G.A. 1999. Maturation, fecundity, and intertidal spawning of Pacific sand lance in the northern Gulf of Alaska. Journal of Fish Biology **54**(5): 1050–1068. doi:10.1111/j.1095-8649.1999.tb00857.x.

Rodomsky, B.T., Kautzi, Li.A., Hannah, R.W., and Good, C. D. 2015. Kelp greenling (*Hexagrammos decagrammus*) growth, spawning seasonality, and female length at maturity based on histological evaluation of ovaries from Oregon waters. Oregon Department of Fish and Wildlife, Marine Resources Program, Newport, Oregon. Available from https://digitalcollections.library.oregon.gov/nodes/view/116424 [accessed 30 April 2025].

Rogers, D.E., Wangerin, M.E., Rogers, B.J., Garrison, K.J., and Rabin, D.J. 1979. Seasonal composition and food web relationships of marine organisms in the nearshore zone of Kodiak Island including ichthyoplankton, meroplankton (shellfish), zooplankton, and fish. Fisheries

Research Institute, Seattle, WA. Available from http://hdl.handle.net/1773/3916 [accessed 30 April 2025].

Serobaba, I.I. 1968. Spawning of the Alaska pollock, *Theragra chalcogramma* (Pallas) in the northeastern Bering Sea. Journal of Ichthyology **8**: 789–798.

Smith, K.R., Somerton, D.A., Yang, M.-S., and Nichol, D.G. 2004. Distribution and biology of prowfish (*Zaprora silenus*) in the northeast Pacific. Fishery Bulletin **102**(1): 168–179.

Smoker, W., and Pearcy, W.G. 1970. Growth and Reproduction of the Lanternfish *Stenobrachius leucopsarus*. J. Fish. Res. Bd. Can. **27**(7): 1265–1275. doi:10.1139/f70-148.

Sohn, D., Ciannelli, L., and Duffy-Anderson, J.T. 2010. Distribution and drift pathways of Greenland halibut (*Reinhardtius hippoglossoides*) during early life stages in the eastern Bering Sea and Aleutian Islands. Fisheries Oceanography **19**: 339–353. doi:10.1111/j.1365-2419.2010.00549.x.

Stark, J.W. 2004. A comparison of the maturation and growth of female flathead sole in the central Gulf of Alaska and south-eastern Bering Sea. Journal of Fish Biology **64**(4): 876–889. doi:10.1111/j.1095-8649.2004.00356.x.

Stark, J.W., and Somerton, D.A. 2002. Maturation, spawning and growth of rock soles off Kodiak Island in the Gulf of Alaska. Journal of Fish Biology **61**(2): 417–431. doi:10.1111/j.1095-8649.2002.tb01574.x.

St-Pierre, G. 1984. Spawning locations and season for Pacific halibut. International Pacific Halibut Commission. Available from https://www.iphc.int/uploads/pdf/sr/IPHC-1984-SR070.pdf.

Turnock, B.J., Wilderbuer, T.K., and Brown, E.S. 1999. Arrowtooth flounder. In Stock assessment and fishery evaluation report for the groundfish resources of the Gulf of Alaska. North Pacific Fishery Management Council, Anchorage, Alaska.
